# Exploring the Nutritional Value and Health Benefits of Honey from the Portuguese Protected Area of Montesinho Natural Park

**DOI:** 10.3390/foods14060963

**Published:** 2025-03-12

**Authors:** Clara Grosso, Sónia Soares, Aurora Silva, Cristina Soares, Manuela M. Moreira, Olena Dorosh, M. Fátima Barroso, Miguel A. Prieto, Cristina Delerue-Matos

**Affiliations:** 1 REQUIMTE/LAQV, Instituto Superior de Engenharia do Porto, Instituto Politécnico do Porto, Rua Dr. António Bernardino de Almeida 431, 4249-015 Porto, Portugal; sefss@isep.ipp.pt (S.S.); mass@isep.ipp.pt (A.S.); cds@isep.ipp.pt (C.S.); mmdsm@isep.ipp.pt (M.M.M.); anelo@isep.ipp.pt (O.D.); mfb@isep.ipp.pt (M.F.B.);; 2 Universidade de Vigo, Nutrition and Food Group (NuFoG), Department of Analytical Chemistry and Food Science, Instituto de Agroecoloxía e Alimentación (IAA)—CITEXVI, 36310 Vigo, Spain; mprieto@uvigo.es

**Keywords:** Montesinho Natural Park, honey, amino acids, proline, phenolic compounds, antioxidant activity, antimicrobial activity, HPLC-DAD, HPLC-FLD

## Abstract

The main objective of this study was to provide an overview of the potential health-promoting properties of honeys collected at specific apiary locations from the protected area of Montesinho Natural Park (MNP), by analyzing their amino acid and polyphenolic profiles, and their biological properties, and comparing them with the renowned Manuka honey. The results revealed differences in total phenolic content among the samples examined and between sampling campaigns, with values ranging from 55.6 to 225 mg gallic acid equivalents (GAE) per 100 g of honey, compared to 57.2 mg GAE/100 g for Manuka honey. Gallic acid, neochlorogenic acid, and catechin were the key phenolics of MNP honey samples. MNP honey exhibited high levels of essential amino acids (phenylalanine, lysine, and valine), strong antioxidant and antimicrobial activities, moderate enzyme inhibition, and high proline content in some locations. These results highlight the exceptional nutritional and therapeutic potential of MNP honey.

## 1. Introduction

The significance of honey in human culture and daily life has been acknowledged since ancient times. Its sustained use across generations is supported by its diverse properties, which offer a wide range of benefits, from nutritional and health-related advantages to applications in food preservation. Honey’s distinctive attributes are determined by its composition, influenced by the geographical and botanical origins of the plants from which bees gather nectar. Environmental and seasonal factors also play a role in shaping these characteristics [1]. The hive’s environment shapes honey’s characteristics, making certain honey high-grade with distinct qualities. Honey from special areas, such as those with Protected Designation of Origin (PDO) status and Manuka honey, are high-quality natural products with distinct physical, chemical, and biological properties. Recently, the demand for exceptional honeys has grown as consumers become more aware of their health benefits [1]. Consequently, research on specific honey has expanded, focusing on quality, safety, and new applications for these biologically enriched products. Among honey’s health benefits, its antioxidant activity stands out. Numerous studies have demonstrated honey’s ability to combat oxidative damage [2,3]. This antioxidant capacity is attributed to compounds found in honey, such as lysozymes, carotenoids, and phenolic compounds, including flavonoids, which act as free radical scavengers [3]. Previous research has linked these compounds to anti-carcinogenic, anti-inflammatory, anti-thrombotic, immune-modulating, and analgesic effects [3]. Additionally, antioxidants like polyphenols, tannins, and flavonoids proved to exhibit enzyme-inhibitory activity [3]. Inactivating certain enzymes can eliminate pathogens or correct metabolic disorders. Specifically, inhibiting angiotensin, tyrosinase, xanthine oxidase, α-amylase, α-glucosidase, acetylcholinesterase, and lipase positively impacts widespread diseases like hypertension, neurodegenerative disorders, and type 2 diabetes [3,4,5]. In that sense, in recent years, natural foods that exhibit inhibitory potential for enzymes have been increasingly incorporated into the human diet. This has led to the development of several studies on inhibitory activity in various natural products, including honey [2].

Research into the antimicrobial potential of honey is also a significant focus within honey-related studies. Honey’s antimicrobial properties are linked to its phenolic compounds, like flavonoids and polyphenols, which act as antioxidants [6]. Additionally, honey’s high osmolarity, low pH (acidity), viscosity (protective barrier), the presence of hydrogen peroxide (H_2_O_2_), non-peroxide components like methylglyoxal, and its ability to create a moist wound environment also contribute to honey’s antimicrobial potential [6,7]. Honey’s preventive properties against wound infections and the inhibitory effects against a wide range of bacterial strains have been extensively studied, highlighting the strong antibacterial effects of certain honeys such as Manuka [6,7,8,9,10].

Aside from its antimicrobial attributes, honey combats infections through various mechanisms, including immune system enhancement, anti-inflammatory and antioxidant effects, and promotion of cellular growth [10]. This is due to its complex composition, which includes health-promoting compounds such as vitamins, minerals, amino acids, enzymes, organic acids, and other constituents. These components, whether acting individually or synergistically, enhance honey’s overall health benefits and effectiveness in promoting health [11].

In Europe, Portugal stands out as the country with the highest number of Protected-Designation-of-Origin (PDO) honeys [8], with the northern region being particularly significant in honey production. Honey originating from the Montesinho Natural Park (MNP), a protected area located in the extreme northeast of Portugal, holds a PDO and is highly esteemed among Portuguese consumers for its distinctive organoleptic qualities and health-related attributes, likely influenced by the park’s exceptional vegetation and climatic conditions [12]. Despite the MNP honey’s high reputation, it is necessary to understand the contribution of the surrounding environment and climatic conditions to its health-related properties.

This study is innovated in providing a comprehensive analysis of the health-promoting properties of Montesinho Natural Park (MNP) honey, comparing its bioactive composition and therapeutic potential with the well-established Manuka honey. By investigating enzyme inhibitory effects, antioxidant capacity, and antimicrobial properties, this research contributes to a deeper understanding of how environmental and botanical factors shape honey’s functional benefits, potentially positioning MNP honey as a valuable natural health product.

## 2. Materials and Methods

### 2.1. Chemicals

Methanol (≥99.8%) used in the extractions and ammonium thiocyanate were obtained from Merck (Darmstadt, Germany). 2,2-Diphenyl-1-picrylhydrazyl radical (DPPH^•^), (±)-6-hydroxyl-2,5,7,8-tetramethylchromane-2-carboxylic acid (Trolox^®^), Folin–Ciocalteu’s (FC) phenol reagent, sodium carbonate (≥99%), Tris(hydroxymethyl)aminomethane (Tris), 5,5′-dithiobis(2-nitrobenzoic acid) (DTNB), acetylthiocholine iodide (ATCI), *S*-butyrylthiocholine iodide (BTCI), galantamine, acetylcholinesterase (AChE) from *Electrophorus electricus*, butyrylcholinesterase (BuChE) from equine serum, tyrosinase from mushroom, 4-nitrophenyl α-D-glucopyranoside, α-glucosidase, bovine serum albumin (BSA), potassium phosphate monobasic (KH_2_PO_4_), potassium phosphate dibasic trihydrate (K_2_HPO_4_.3H_2_O), β-nicotinamide adenine dinucleotide (NADH) disodium salt hydrate, phenazine methosulphate (PMS), nitrotetrazolium blue chloride (NBT), sodium nitroprusside dihydrate (SNP), sulphanilamide, naphthylethylenediamine dihydrochloride, ortho-phosphoric acid 85%, L-DOPA (L-3,4-dihydroxyphenylalanine), sodium hydroxide, salicylic acid, iron(III) chloride (97%), and potassium hexacyanoferrate(II) trihydrate (≥99%) were purchased from Sigma-Aldrich (St. Louis, MO, USA, and Steinheim, Germany). 2,4,6-Tris(2-pyridyl)-s-triazine (TPTZ, 99%), (−)-epicatechin (≥90%), gallic acid (GA, ≥99%), and hydrochloric acid (≥37%) were obtained from Fluka (Munich, Germany). Aluminum chloride (99.52%) and sodium acetate 3-hydrate (99%) were acquired from Panreac (Barcelona, Spain). Iron(III)chloride-6-hydrate (≥99%) and L(+)-ascorbic acid (AA, ≥99.7%) were from Riedel-de Haën (Seelze, Germany). Glacial acetic acid (≥99.5%), pH Meter Calibration Solutions (pH 4 and pH 7), and hydrogen peroxide (30%) were from Carlo Erba (Peypin, France); sodium nitrite (98%) was from M&B Chemicals (City Road, London, the UK), and sodium hydroxide (>99%) was from Labkem (Zelienople, PA, USA).

For HPLC analysis, methanol (≥99.8%) and formic acid (≥98%) were gradient-grade and from Merck (Darmstadt, Germany). The standards of phenolic compounds (described in Supplementary Materials) were purchased from Sigma-Aldrich and Extrasynthèse. Their individual stock solutions were prepared in HPLC gradient-grade methanol at concentrations from 1 to 5 g/L, which were used to prepare the mixtures of solutions for the calibration curves.

Magnesium chloride hexahydrate was obtained from VWR (Leuven, Belgium), sodium phosphate dibasic anhydrous (Na_2_HPO_4_) was obtained from Carlo Erba (Val de Reuil, France), and sodium chloride was obtained from Fisher Scientific (Fair Lawn, NJ, USA). Ultrapure water (resistivity of 18.2 MΩ·cm at 25 °C) was produced using a Simplicity 185 system (Millipore, Molsheim, France).

For amino acid analysis, the standards (described in Supplementary Material Table S3) were sourced from Sigma-Aldric, Fluka, and Merck. The individual stock solutions were prepared at a concentration of 1000 mg/L in 0.1 M HCl solution (Carlo Erba). Ortho-phthalaldehyde (OPA), fluorenylmethyloxycarbonyl chloride (FMOC-Cl), 3-mercaptopropionic acid (3-MPA), and sodium acetate were acquired from Sigma. Boric acid was from VWR (Radnor, PA, USA), triethylamine (TEA) was from Carlo Erba, acetic acid was from Panreac, acetonitrile was from Chromasolv, and HPLC gradient-grade methanol was from Honeywell (Charlotte, NC, USA). Nylon syringe filters (0.22 µm) were acquired from Specanalitica (Carcavelos, Portugal).

### 2.2. Sampling

A total of 8 apiaries with different locations within the MNP were previously selected, aiming to include a wide range of territories within the park. The MNP is predominantly characterized by schist soils but also features granite, ultrabasic rocks, and small limestone patches. The flora is extremely diverse due to the combination of geological and climatic factors. It includes oak forests (*Quercus pyrenaica* Willd.); chestnut groves (*Castanea sativa* Mill.); Holm oak woods (*Quercus rotundifolia* Lam.); broom scrublands (dominated by species such as *Cytisus scoparius* (L.) Link, *Cytisus striatus* (Hill) Rothm., and *Cytisus multiflorus* (L’Hér.) Sweet); heathlands (including *Erica australis* ssp. *aragonensis* (Willk.) Samp and *Erica tetralix* L.); rockrose scrublands (mainly composed of *Cistus ladanifer* L. and *Lavandula stoechas* L. ssp. *sampaiana*); meadows (rich in floristic species such as *Serapias lingua* L., *Dactylorhiza maculata* (L.) Soó, *Paradisea lusitanica* (Cout.) Samp., *Ajuga pyramidalis* ssp. *meonantha* L., and *Thymus pulegioides* L.). In the ultrabasic soils, rare and endemic plants occur, such as *Armeria eriophylla* Willk., *Anthyllis sampaiana* Rothm., and *Avenula pratensis* ssp. *Lusitanica* Romero Zarco, unique species that are not found anywhere else in the world (https://www.icnf.pt/conservacao/rnapareasprotegidas/parquesnaturais/pnmontesinho, accessed on 1 March 2025).

The honey samples included in this work were obtained directly from local producers of the corresponding apiaries and could be divided into two sets: the first set of samples, comprising 8 honey samples, was obtained in 2021 (1st campaign). These honey samples were included in a previous work aiming for its comprehensive characterization regarding the main physicochemical properties [12]; the second set of samples, comprising 8 honey samples, was obtained in 2022 (2nd campaign). Both campaigns were conducted between August 20 and September 20. According to the meteorological report for 2021, the average air temperature during this period ranged from 22 to 18 °C, with precipitation varying from 10 to 20 mm between August and September. In the second campaign (2022), the average air temperature ranged from 24 to 18 °C, while precipitation varied from 5 to 50 mm between August and September. (https://www.ipma.pt/pt/publicacoes/boletins.jsp?cmbDep=cli&cmbTema=pcl&cmbAno=2021&idDep=cli&idTema=pcl&curAno=2021, accessed on 1 March 2025).

The locations of the first set of samples correspond to those of the second set, resulting in a total of 16 honey samples (Table S1, Supplementary Materials). All the samples were multifloral honeys. Honey samples were immediately transported to the laboratory, stored at room temperature in the dark and analyzed, at maximum, after six months. Additionally, one commercial sample labeled as Manuka honey (COMVITA^®^, MGO* 83+, UMF 5+, Paengaroa, New Zealand) was purchased in a local supermarket.

### 2.3. Ultrasound-Assisted Extraction (UAE)

The ultrasound extraction process, as detailed by Bayomy et al. [13], was followed with slight modifications. Honey samples were prepared by homogenizing 1 g of honey and 10 mL of methanol in 15 mL conical centrifuge tubes. The tubes were placed in an ultrasound bath at 45 °C for 20 min. After extraction, the samples were centrifuged (Heraeus Megafuge 16 Centrifuge Series, Thermo Scientific, Waltham, MA, USA) at 8000 rpm for 10 min, and the supernatant was stored in a freezer (at −20 °C) until further use. All extractions were performed in duplicate.

### 2.4. Determination of the Total Phenolic and Flavonoid Content

The total phenolic content (TPC) assay was based on the method originally reported by Singleton and Rossi [14] and performed as described in detail by Dorosh et al. [15]. The reaction consists of the Folin–Ciocalteau reagent changing color from yellow to blue after reacting with reducing species under alkaline conditions. A standard curve of GA was established, and the results were expressed as mg of GA equivalents (GAE) per 100 g of honey (mg GAE/100 g honey). Each UAE methanolic extract was analyzed in triplicate.

The total flavonoid content (TFC) method measures the formation of the flavonoid-aluminium compound and was performed according to Dorosh et al. [15]. Epicatechin was used as a standard, and the results were expressed as mg epicatechin equivalents (EE) per 100 g of honey (mg EE/100 g honey). Each UAE methanolic extract was analyzed in triplicate.

### 2.5. Determination of the Phenolic Composition by HPLC-DAD

According to our previous report, the phenolic composition of UAE methanolic extracts was determined by high-performance liquid chromatography with a diode-array detector (HPLC-DAD) [16]. Before analysis, honey extracts were filtered (0.22 mm PTFE membrane filters), and the phenolic compounds were separated at 25 °C using a Gemini C_18_ column (250 × 4.6 mm, 5 μm particle size) from Phenomenex (Torrance, CA, USA). The mobile phase consisted of water (solvent A) and methanol (solvent B), both acidified with 0.1% formic acid. The system was run with the following gradient elution program: 0–5 min: 20–24% A; 5–7 min: 24–25% A; 7–10 min: 25–26% A; 10–11 min: 26–26.5%A; 11–18 min: 26.5% A; 18–25 min: 26.5–30% A; 25–50 min: 30–45% A; 50–60 min: 45–50% A; 60–70 min: 50–55% A; 70–90 min: 55–70% A; 90–100 min: 70–100% A. A 5 min post-run at initial conditions for equilibration of the column was performed before the next injection. The flow rate was kept constant throughout the analysis at 1 mL/min, and the injection volume was 20 µL. The retention times and UV-Vis spectra were compared with those of pure standards for identifying individual phenolic compounds. Quantification was carried out using wavelengths of 280, 320, and 360 nm based on the maximum absorption of the identified phenolic compounds. Relevant analytical data, namely regression equations, limit of detection (LOD), and quantification (LOQ), are shown in Table S2 (Supplementary Materials). The results were expressed as mg of phenolic compound per 100 g of honey (mg/100 g honey) of three determinations (n = 3).

### 2.6. Determination of the Amino Acids Profile

Honey samples were prepared by dissolving 3 g in 25 mL of ultrapure water. After homogenization in a vortex, samples were filtered through a syringe filter [17]. All samples were prepared in duplicate. These samples were also used for pH analysis and hydrogen peroxide determination.

Amino acids were analyzed using HPLC Shimadzu system (Tokyo, Japan), which included an SCL-40 system controller, an LC-40D pump, a CTO-40C column oven, a four-channel low-pressure gradient unit, a SIL-40C automatic injector, a DGU-405 5-channel degasser, and an RF-20Axs fluorescence detector (FLD). The analysis of amino acids by HPLC-FLD involved a derivatization step. The derivatization of the samples and standards was carried out in a 1.5 mL amber vial using the following procedure: 80 µL of the sample or standard was mixed with 200 µL of the internal standard solution (norvaline), and 200 µL of borate buffer (80 mM boric acid, pH 10.4), 80 µL of the OPA/3-MPA derivatizer, and 8 µL of the FMOC-Cl derivatizer were dissolved in acetonitrile [18]. The derivatized compounds were separated on a Kinetex Core-Shell C18 column (5 µm, 150 mm × 4.6 mm, Phenomenex, Torrance, CA, USA) with a C18 pre-column (Phenomenex). The mobile phase was prepared using a gradient of two solutions: solution A (25 mM acetate buffer solution, prepared by dissolving 2.05 g of sodium acetate in 1 L of water and adding 0.05% TEA, adjusted to pH 7.2 with concentrated acetic acid) and solution B (a mixture of water, acetonitrile, and methanol in a 20:40:40 ratio, *v*/*v*).

The chromatographic conditions used to analyze amino acids by HPLC-FLD are detailed as follows: the gradient conditions were set as 0 min at 100% A, 27 min at 60% A, 30 min at 0% A, 32 min at 100% A, and 35 min at 100% A. The flow rate was maintained at 1.0 mL/min, with an injection volume of 10 µL. The column oven temperature was set to 42 °C. Fluorescence detection involved the use of amino acid OPA-3MPA derivatives with excitation at 340 nm and emission at 450 nm and the use of FMOC-Cl derivatives with excitation at 237 nm and emission at 340 nm, providing higher sensitivity for proline. All samples were injected in duplicate. Calibration parameters are presented in Table S3 (Supplementary Materials).

The limits of detection and quantification were calculated using the following equations:

LOD = (3 × SD_blank_)/m and LOQ = (10 × SD_blank_)/m, where SD_blank_ represents the standard deviation of the blank, and m is the slope of the straight-line equation. The LODs ranged from 0.179 μg/L for Val to 22.9 μg/L for Lys, and the LOQs ranged from 0.597 for Val to 76.2 μg/L for Lys.

### 2.7. pH Determination

The pH of the aqueous honey solutions, prepared by dissolving 3 g of sample in 25 mL of ultrapure water, was measured using a multiparameter analyzer (Consort C861, Turnhout, Belgium) equipped with a combined pH electrode (Consort SP10B). The electrode was calibrated using standard pH 4 and pH 7 buffer solutions, then rinsed with distilled water and dried before measuring the pH of the honey samples.

### 2.8. Hydrogen Peroxide Determination

The hydrogen peroxide determination was performed based on the method described by Branco et al. [19], using a modified IDF method based on the oxidation of Fe(II) to Fe(III) by hydroperoxides and the subsequent formation of an Fe(III)-thiocyanate complex for spectrophotometric analysis.

Briefly, 3 g of honey was dissolved in 25 mL of ultrapure water. A 2 mL aliquot of this solution was mixed with 10 μL of ammonium thiocyanate solution and vortexed for 15 s. Subsequently, 10 μL of Fe(II) solution was added, and 180 μL of the final mixture was transferred to a microplate. Absorbance was measured at 500 nm. A reaction blank was included for background correction. The calibration curve was prepared using six Fe(III) standards with concentrations ranging from 0.035 to 0.20 μg/mL. Results were expressed as H_2_O_2_ in μg/100 g of honey.

### 2.9. Bioactivities

#### 2.9.1. Assessment of the In Vitro Antioxidant Activity

Several complementary methods evaluated the antioxidant activity of the honey samples. The ferric-reducing antioxidant power (FRAP) of samples was based on the reduction of a ferric complex (Fe^3+^-TPTZ) to the ferrous form (Fe^2+^-TPTZ) after reacting with antioxidants. This assay was performed according to the protocol reported by Benzie and Strain [20], with some minor modifications as described by Dorosh et al. [15]. Ascorbic Acid (AA) was used as a standard, and the results were expressed as milligrams of AA equivalents (AAE) per 100 g of honey (mg AAE/100 g honey). Each UAE methanolic extract was analyzed in triplicate.

The DPPH-radical scavenging activity (DPPH-RSA) assay evaluated the sample’s capacity to reduce DPPH^•^ to hydrazine, and it was performed as described by Dorosh et al. [15]. Trolox was used as a standard, and the results were expressed in milligrams of Trolox equivalents (TE) per 100 g of honey (mg TE/100 g honey). Each UAE methanolic extract was analyzed in triplicate.

The ABTS-RSA assay is based on antioxidants reducing the ABTS^•+^. It was performed according to Re et al. [21] with minor modifications as described by Dorosh et al. [15]. Trolox was used as a standard, and ABTS values were expressed as mg TE/100 g honey. Each UAE methanolic extract was analyzed in triplicate.

The scavenging activities of superoxide anion radical (O_2_^•−^) and nitric oxide (^•^NO) were assessed following the methodology of Soares et al. [22]. For O_2_^•–^ scavenging activity, honey samples were dissolved in a 19 mM potassium phosphate (KH_2_PO_4_/K_2_HPO_4_) buffer (pH 7.4). The O_2_^•−^ radical was generated using the NADH/PMS system, with potassium phosphate buffer as the negative control. Blanks included all reagents except PMS, and kinetic curves were recorded at 560 nm using a Synergy HT microplate reader (BioTek Instruments, Winooski, VT, USA). For ^•^NO scavenging activity, honey samples were dissolved in a 0.1 M KH_2_PO_4_/K_2_HPO_4_ buffer (pH 7.4) and incubated with sodium nitroprusside (SNP) for 60 min (room temperature, under light) as described previously [22]. Post-incubation, Griess reagent was added to each well. The mixture was then incubated at room temperature for 10 min, and the absorbance was measured at 560 nm using the BioTek Synergy HT microplate reader. The phosphate buffer was the negative control, and 2% phosphoric acid was added to the blanks instead of the Griess reagent. All assays were performed in triplicate, and results were expressed as IC_50_ values. Hydroxyl radical scavenging (^•^HO) was performed based on the salicylic acid method as described formerly [23], with some modifications [24]. Honey samples were dissolved in demineralized water, blanks were prepared by replacing H_2_O_2_ with demineralized water, and negative controls were prepared by substituting samples with water. Absorbances were recorded at 510 nm. Seven sequential dilutions were made and tested in triplicate. Results were expressed as IC_50_ values.

#### 2.9.2. Enzyme Inhibition Activity

For enzyme inhibition assays, samples were tested in the range of 2.00–0.03 mg/mL. AChE and BuChE inhibition was assessed according to the Ellman method with minor modifications [22]. Briefly, honey samples were dissolved in Tris-HCl buffer (50 mM, pH = 8) and added to the wells, along with the DTNB reagent, buffer B (Tris-HCl buffer + 0.1% albumin), ATCI/BTCI, and AChE or BuChE solutions. Slopes were calculated from the kinetic curve obtained at 405 nm, after subtracting the blanks (wells containing all reagents except the enzyme). The Tris-HCl buffer was used as a negative control.

Tyrosinase inhibition was measured according to Masuda et al. [25]. Honey samples were dissolved in 1/15 M of Na_2_HPO_4_/KH_2_PO_4_ (pH = 6.8), and the solution was added to the wells (or buffer for a negative control) followed by the addition of the buffer and tyrosinase solution. After 10 min of incubation at room temperature, L-DOPA was added. After another 10 min, the absorbance was measured at 475 nm. L-DOPA was replaced by buffer solution in blanks.

α-Glucosidase inhibitory activity was assessed as previously reported [26]. In brief, the reaction mixture consisted of 4-nitrophenyl α-D-glucopyranoside dissolved in 10 mM potassium phosphate buffer (pH 7.0), phosphate buffer, and honey samples dissolved in buffer or buffer alone (for the negative control). The reaction was initiated by adding the enzyme solution. The kinetic was traced at 405 nm for 10 min at 37 °C. All enzymatic assays were performed in triplicate.

#### 2.9.3. Assessment of the Antimicrobial Activity

The MIC (minimum inhibitory concentration) and MBC (minimum bactericidal concentration) were determined according to the previously described method [27] with one modification: the MBC was determined by plating 10 µL of the samples from the wells (at least duplicates) in which no bacterial growth was visible onto Muller-Hinton agar plates and incubating for 24 h. The absence of colonies determined the MBC.

The microorganisms tested were provided by Selectrol, Buckingham, the UK: *Staphylococcus aureus* (ATCC 25923) and *Bacillus cereus* (ATCC 14579), *Pseudomonas aeruginosa* (ATCC 10145), and *Salmonella enteritidis* (ATCC 13076), while *Staphylococcus epidermidis* (NCTC 11047) and *Escherichia coli* (NCTC 9001) were supplied by Microbiologics (St. Cloud, MN, USA). The maximum concentration tested was 37.5%.

### 2.10. Statistical Analysis

All statistical analysis was performed with the software GraphPad Prism 8.0.1. Comparisons between MNP and Manuka samples were performed using one-way analysis of variance (ANOVA) followed by the Tukey’s test, and *p*-values less than 0.05 were considered to be statistically significant. Pearson correlation analyses were carried out to determine the possible relations between bioactive compounds quantified and the observed bioactivities.

## 3. Results and Discussion

### 3.1. Phenolic Content

Table 1 summarizes the TPC and TFC values of the honey samples from MNP collected in 2021 (1st campaign) and 2023 (2nd campaign). For the 1st sampling campaign, the TPC ranged between 55.58 ± 0.89 and 72.77 ± 1.54 mg GAE/100 g honey for MNP8 and MNP2 samples, respectively. In the case of the 2nd sampling campaign, the TPC ranged from 62.16 ± 2.93 to 225.43 ± 1.89 mg GAE/100 g honey for MNP5 and MNP2 samples, respectively. Excluding the MNP2 sample from the 2nd sampling campaign, the average TPC values were similar, corresponded to 67.40 and 69.20 mg GAE/100 g honey for the 1st and 2nd sampling campaigns, respectively. These values are higher than those found for the Manuka sample (57.16 mg GAE/100 g honey). Several authors [28,29,30,31,32] evaluated the phenolic and flavonoid content in Portuguese honey, reporting similar values when compared to the present study. Ferreira et al. [32] analyzed three uni-floral honeys, also from MNP, reporting a TPC ranging from 22.6 to 72.8 mg GAE/100 g honey.

The broader TPC range reported by these authors indicates that the botanical origin of honey can exert a significant impact on its phenolic content. Soares et al. [29] analyzed 15 Portuguese honeys from different botanical and geographical origins and the lowest TPC value was reported for lavender honey, while the highest value corresponded to heather honey (14.9 and 50.9 mg GAE/100 g honey, respectively).

These results agree with the ones from Ferreira et al. [32], demonstrating that TPC depends not only on the botanical origin of the samples but also on the geographical origin. In fact, three of the honey samples analyzed by Soares et al. [29] were multi-floral, and the obtained values were lower than 37.9 mg GAE/100 g honey, demonstrating that multi-floral honey produced in different regions generally present lower amounts of phenolics. In another study, Estevinho et al. [31] determined the TPC and TFC from 75 honey samples from different organic apiaries in Trás-os-Montes region and concluded that sampling locations within the same region highly influence the presence of differences in phenols and flavonoids levels.

**Figure 1:**
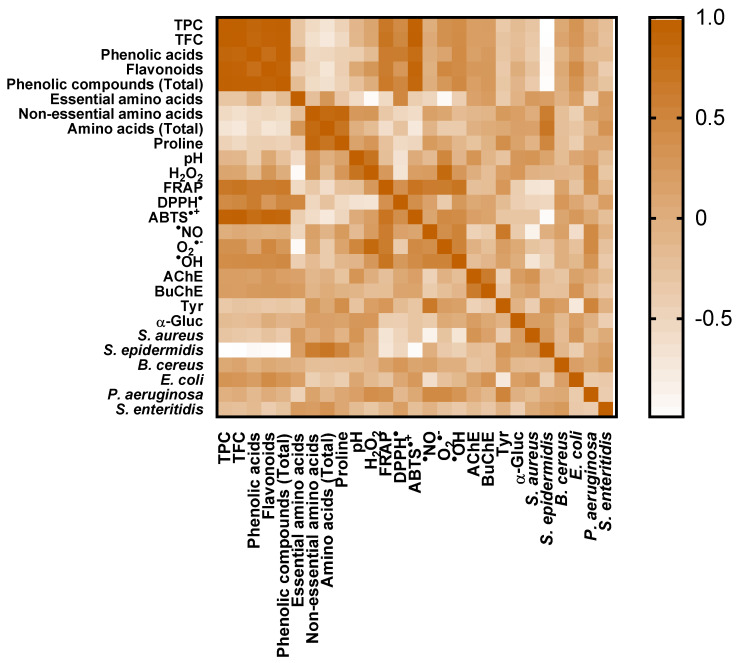
Heatmap of the Pearson correlation coefficient matrix.

Recently, Zivkovic et al. [33] described that natural honey is one of the highly appreciated and valued flavonoid food sources. In the present study, TFC values for both sampling campaigns ranged from 3.70 ± 0.18 mg EE/100 g honey (MNP7, 2nd campaign) to 35.2 ± 0.6 mg EE/100 g honey (MNP2, 2nd campaign), with Manuka sampling containing 3.52 mg EE/100 g honey. These results were consistent with Zivkovic et al. [33], who reported TFC values ranging from 3.16 to 16.2 mg CE/100 g. In contrast, Estevinho et al. [31] reported TFC ranging from 49.4 to 56.3 mg CE/100 g in organic honey from Trás-os-Montes region, which were at least 6-fold higher than the values determined in this study. As previously observed for TPC, the mean TFC values between honey samples and sampling campaigns were similar (5.49 and 5.17 mg EE/100 g honey for the 1st and 2nd sampling campaigns, respectively). The correlation between the two spectrophotometric assays was high (R < 0.986) as the honey samples that presented the highest TPC were the ones with the highest TFC (Figure 1).

According to Soares et al. [29], the observed differences in TPC and TFC values among the analyzed samples can be attributed to the geographical origin of the honey. In this study, although all samples were collected from MNP, they came from different locations within the park, which may justify the obtained differences. Some authors [31,34,35] reported that various plants contain diverse phenolics and flavonoids present in different amounts, depending on their species as well as climatic and soil conditions. Indeed, these factors can vary within the same sampling region, thereby affecting the bioactive properties of honey samples.

Comparing the obtained results from honey samples from MNP with the honey from other countries, a huge diversity in phenolic and flavonoid content was observed [13,34,36,37]. Cheung et al. [37] analyzed 40 honey samples produced in China and imported from other countries, reporting TPC and TFC lower than the levels found in the present study (24.5 and 2.3 mg/100 g honey for TPC and TFC, respectively). The variation in the results obtained was most likely due to differences in botanical and geographical origin. Kavanagh et al. [34] reported that TPC values for Irish honey ranged from 2.59 to 81.1 mg GAE/100 g and suggested that these differences could be linked to the landscape context, particularly the main type of land use around the sampled hives. Considering the results obtained, these authors concluded that the flower origin has a crucial impact on the TPC value. The great variation between the multi-floral honey is influenced by the main flower species present in the landscape corresponding to each honey sample. Pedisić et al. [36] analyzed mono-floral honey samples from different geographical regions of Croatia, reporting TPC values ranging from 55.9 to 87.9 mg GAE/100 g. Similarly, Bayomy et al. [13] examined Egyptian honey from eleven botanical origins, with TPC values ranging from 37.2 to 138 mg GAE/100 g. Both studies reported TPC ranges higher than the levels found in the present study. All these studies highlight the influence of botanical and geographical origins on honey’s phenolic and flavonoid contents [34].

### 3.2. Phenolic Composition by HPLC-DAD

The HPLC analysis of honey samples, as detailed in Table 2, reveals that the most abundant phenolic compounds in MNP samples are gallic acid, neochlorogenic acid, and catechin. On the other hand, the profile of Manuka honey was different, showing higher gallic acid content than MNP samples but reduced amounts of neochlorogenic acid and catechin. These three compounds were present in all MNP samples for both sampling campaigns. Phenolic acids were in higher concentration than flavonoids, representing at least 40% of the total phenolic compounds quantified.

The total identified phenolic compounds from the 1st campaign samples ranged from 30.02 to 57.86 mg/100 g of honey, while for the 2nd campaign, it ranged from 36.45 mg/100 g (MNP5) to 158.76 mg/100 g (MNP2). For the Manuka sample, the obtained value was 45.87 mg/100 g of honey. Gallic acid was one of the most dominant phenolic acids in the honey samples, ranging from 3.31 ± 0.16 to 9.68 ± 0.48 mg/100 g, while Manuka honey contained 37.14 mg/100 g. Within the same botanical origin but different apiary locations, the MNP4 sample (9.68 ± 0.48 mg/100 g) possessed much higher gallic acid content than the MNP6 honey (5.25 ± 0.26 mg/100 g) both from the 1st campaign. Gallic acid values from the 1st campaign were at least 1.5-fold higher than those obtained for the 2nd sampling campaign. Previous studies have also reported a high gallic acid content in many honeys [37,38]. Cheung et al. [37] determined the phenolic profile from 40 commercial honey samples from different country origins and reported gallic acid contents below 3.87 mg/100 g. In addition, gallic acid was reported as the main compound in Turkish honey, with contents like honey samples from MNP (2.56 to 12.5 mg/100 g) [38]. These studies demonstrate the vast diversity of gallic acid content in honey samples with different botanical and geographical origins.

Protocatechuic acid was also one of the most predominant hydroxybenzoic acids in honey samples studied, with values ranging from 1.25 ± 0.06 in the MNP3 sample to 14.9 ± 0.75 mg/100 g in the MNP2 sample from the 2nd campaign. From the 40 honey samples analyzed by Cheung et al. [37], this phenolic acid was only quantified in seven kinds of honeys, with levels below 6.36 mg/100 g honey. Hernanz et al. [39] also report the presence of protocatechuic acid in oak honey samples collected in diverse Spanish regions, and the maximum value was 0.435 mg/100 g, which was at least 3-fold lower than the present values. Further, the levels found for vanillic acid in honey samples from MNP must also be highlighted as it represents a phenolic acid not commonly identified in honey. Indeed, Cheung et al. [37] only reported the presence of this compound in one honey from a total of 40 samples analyzed (0.51 mg/100 g). Küçükaydın et al. [38] also reported the presence of this hydroxybenzoic acid in the 37 Turkish honeys analyzed; however, the maximum amount found was 0.45 mg/100 g, which was lower than the levels reported herein. 

The second dominant phenolic acid in MNP samples from both campaigns was neochlorogenic acid, ranging from 1.49 ± 0.07 mg/100 g (MNP6 sample, 1st sampling campaign) to 14.77 ± 0.74 mg/100 g (MNP2 sample, 2nd sampling campaign). Despite all samples being collected in apiaries from the same region, the neochlorogenic acid levels were different. Chlorogenic and caffeic acids have also been reported to be common hydroxycinnamic acids in honey samples [36,37,39], with demonstrated antioxidant activity. Both compounds were identified and quantified in honey from MNP, but their content was much lower than neochlorogenic acid, except for the MNP2 sample from the 2nd campaign (20.09 ± 1.00 mg/100 g). In a previous study [37], multifloral honeys from Spain and Italy reported similar contents of chlorogenic (1.96 mg/100 g) and caffeic acids (2.09 mg/100 g). In Hernanz et al.’s study [39], the levels found for chlorogenic and caffeic acids (6.63 and 0.48 mg/100 g, respectively) were also in agreement with the ones from the present study. These results indicate that honey from similar geographical locations and floral sources can still exhibit variations in the composition and content of phenolic acids [13,37].

From the identified and quantified flavonoid compounds, it should be noted that some of them, such as isorhamnetin-3-*O*-glucoside, kaempferol-3-*O*-rutinoside, and naringenin, were only found in the MNP4 sample from the 1st sampling campaign. Phloretin was only observed in the MNP2 and MNP6 samples (0.59 ± 0.03 and 0.123 ± 0.006 mg/100 g, respectively) from the 2nd campaign. Among the other flavonoids, catechin, quercetin-3-*O*-galactoside, and phloridzin were the most common and frequently found compounds in honey samples from both sampling campaigns. At least 22 flavonoid compounds were identified and/or quantified in the honey samples from MNP. Still, for some of them, the concentration was too low to be quantified.

The most abundant flavonoid among all honey samples was catechin (from 3.05 ± 0.15 mg/100 g in MNP4 honey to 42.56 ± 2.13 mg/100 g in MNP2 honey, 2nd campaign). The second most abundant flavonoid was quercetin-3-*O*-galactoside, with levels ranging from 1.20 ± 0.06 mg/100 g in MNP2 honey to 6.53 ± 0.33 mg/100 g in MNP3 honey from the 1st campaign. It should be mentioned that, in the case of the 2nd sampling campaign, this compound was below the limit of detection in the MNP2 sample, which was surprising as this honey sample presented the highest amount of total phenolics quantified (158.76 mg/100 g). Phloridzin, a dihydrochalcone, was the third main flavonoid in MNP honeys, varying from 1.33 ± 0.07 mg/100 g in MNP8 honey from the 1st campaign to 5.25 ± 0.26 mg/100 g in MNP3 honey from the 2nd campaign (Table 2). A high content of epicatechin was also detected in MNP7 honey from the 1st campaign (4.15 ± 0.21 mg/100 g) followed by MNP5 (1.22 ± 0.06 mg/100 g), while the levels in other honey samples were below 0.40 mg/100 g or not detected as in the MNP4 sample. In the 2nd campaign, epicatechin amounts ranged from 0.28 ± 0.01 for MNP3 to 3.84 ± 0.19 mg/100 g for MNP2. In the MNP4 sample, the epicatechin amount was 1.98 ± 0.09 mg/100 g, suggesting that the year of sampling can influence the type and the number of phenolic compounds detected in MNP honey.

**Figure 2:**
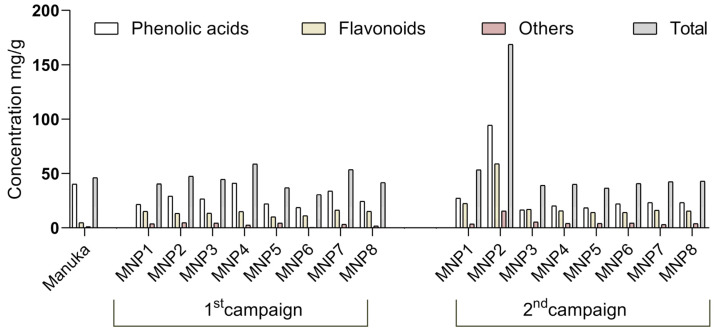
Main classes of phenolic compounds identified in MNP and Manuka honeys.

In the case of the flavanone naringenin, for samples MNP2, MNP4, and MNP7 from the 1st campaign, the levels were higher than 2.46 mg/100 g, while for the other samples, the levels were below the limits of detection or quantification. In the 2nd campaign, the naringenin level stood out in the MNP2 sample (5.60 ± 0.28 mg/100 g), while in the other samples, the levels were at least 3-fold lower. For naringin, in the 1st sampling, the levels varied between 0.15 ± 0.01 and 0.56 ± 0.03 mg/100 g, while in the 2nd sampling, the amount observed was similar except for the MNP1 sample (1.67 ± 0.08 mg/100 g). The total content of phenolic acids, flavonoids, and other phenolic compounds identified in MNP and Manuka honeys is shown in Figure 2.

Other authors previously reported the presence of these compounds [36,37]. From the 40 honey samples analyzed by Cheung et al. [37], these flavonoids were only detected in 10 of them, and the highest levels were 1.92 and 0.21 mg/100 g for naringin and naringenin, respectively. The same authors also reported that chrysin was the third main flavonoid in their honey samples. In the present study, despite the lower values of chrysin compared to the other flavonoids identified, the levels found in MNP samples were at least 10-fold higher than the amount reported by Cheung et al. [37]. Pedisić et al. [36] also reported chrysin as one of the most abundant in their honey samples, with levels ranging from 0.165 to 0.981 mg/100 g, which agrees with values observed in the present study, except for MNP7 honey from the 1st sampling (1.31 ± 0.07 mg/100 g). Myricetin, commonly found in honey samples, was also detected in MNP honeys; however, differences were observed between both campaigns, with honey from the 2nd sampling presenting at least 2-fold higher amounts. Rutin was also identified in all samples from the 2nd sampling campaign (0.88 ± 0.04 to 2.72 ± 0.14 mg/100 g), while honey samples from the 1st campaign did not contain this compound. Both flavonols were found in mono-floral honey samples analyzed by Pedisić et al. [36], which reported the content ranges of 0.016 to 0.912 mg/100 g and 0.023 to 0.226 mg/100 g for myricetin and rutin, respectively.

Generally, most phenolic compounds identified and quantified in MNP samples have previously been found in other mono- and multifloral honey from different origins, although in varying amounts [36,37,39]. This suggests that botanical and geographical origins, along with the sampling year, location, and factors like extraction methods, applied techniques, packaging, storage, and analytical tools, may significantly affect honey’s phenolic composition and content.

### 3.3. Amino Acids Profile

Table 3 focuses on the amino acid content of MNP honey samples from the 1st and 2nd campaigns. The results were also compared with a commercial sample of Manuka honey. Proline content was particularly emphasized, with extremely high levels, particularly in MNP3 (116.44 mg/100 g, 2nd campaign), MNP4 (155.10 mg/100 g, 1st campaign), and MNP7 (135.36 mg/100, 2nd campaign). Proline is a key indicator of honey quality related to antioxidant activity, maturity, and natural provenance [40]. A minimum value of 18 mg of proline/100 g is accepted as the limit for pure honey [41]. At the same time, some authors reported proline levels in honey from 50 to 85% of the total amino acid content [40]. MNP honey exhibited proline levels ranging from 31.08 to 59.20%. However, it is important to note that proline levels can vary significantly depending on the type of honey studied [42]. Thus, the high proline content confirms that MNP honeys are of high quality and maturity, which is preferred for therapeutic applications. Proline is essential for collagen formation, wound healing, and immune function [43]. Manuka honey has a proline content of 42.12 mg/100 g, lower than the highest levels found in MNP honeys (e.g., MNP4 with 155.10 mg/100 g, 1st campaign). This indicates that some MNP honey samples are of high quality, surpassing even the renowned Manuka honey analyzed in the present study. 

Table 3 shows that the essential amino acid phenylalanine concentration was consistently high across several spots, with significant concentrations in MNP1 (28.30 mg/100 g), MNP4 (21.28 mg/100 g), and MNP7 (25.73 mg/100 g) from the 2nd campaign. This indicates important protein content, which benefits cognitive functions and mental health [44].

There were also high concentrations of glutamic acid in MNP3 (22.71 and 22.73 mg/100 g in the 1st and 2nd campaigns, respectively), MNP2 (20.46 mg/100 g in the 1st campaign) and MNP1 (19.51 mg/100 g in the 2nd campaign). Glutamic acid is important for metabolism and brain function, enhancing the nutritional value of honey [45]. Manuka honey is widely known for its high medicinal value. The results obtained for MNP honey samples, particularly from spots like MNP4 and MNP7, demonstrate competitive or superior levels of key amino acids, suggesting similar or enhanced nutritional and therapeutic benefits.

Considering the variability between collection spots, MNP7 honey consistently showed high levels of proline and other amino acids across both sampling years, suggesting an optimal environment for high-grade honey production. MNP1 and MNP4 also showed significant concentrations of key amino acids, like phenylalanine and proline, indicating robust floral diversity and favorable environmental conditions for honey maturity. MNP2 and MNP5 spots exhibited lower amino acid concentrations in the 2nd campaign than in the 1st campaign, which may reflect environmental changes or variations in floral availability [46].

**Figure 3:**
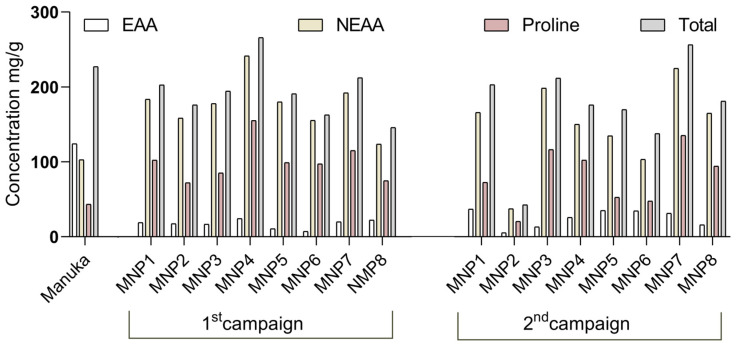
Total content of essential and non-essential amino acids and proline in MNP and Manuka honeys.

The diverse amino acid profile, particularly the presence of essential amino acids (Table 3, Figure 3), highlights the nutritional value of MNP honeys. Essential amino acids such as phenylalanine and lysine, and non-essential amino acids such as glutamic acid are critical for various bodily functions, including protein synthesis, enzyme production, and cognitive health [45]. The variability in amino acid content across different collection spots provides valuable insights into the ecological diversity of MNP and its impact on honey production. This variability can be used to identify the best locations for high-quality honey production and understand environmental factors’ influence on honey composition [47].

### 3.4. pH and H_2_O_2_ Determination

The pH values of MNP honeys are displayed in Table 1, with values ranging from 3.23 (Manuka honey) to 5.03 (MNP5, 2nd campaign). These values are in accordance with those obtained for other Portuguese honey samples. Machado et al. [48] analyzed 153 kinds of honey from different botanical sorigins produced in different parts of mainland Portugal and the Azores archipelago. The obtained values were within pH 3.2 (e.g., lavender and rape honeys) and 4.4 (e.g., chestnut and strawberry tree honeys).

Hydrogen peroxide (H_2_O_2_) is a significant component in many types of honey, contributing to its antimicrobial properties [49]. When bees add glucose oxidase to nectar, it converts glucose into gluconic acid and H_2_O_2_ [50]. This slow-release H_2_O_2_ acts as a mild antiseptic, contributing to the antimicrobial efficacy of honey against various bacteria, including both Gram-positive and Gram-negative strains [51]. Honey’s H_2_O_2_ disrupts bacterial cell walls and inhibits enzyme systems, effectively killing or inhibiting bacteria growth, thus making it effective in preventing wound infections and promoting faster healing [52]. Hydrogen peroxide in honey helps reduce inflammation at wound sites by controlling bacterial infections and reducing oxidative stress [52].

H_2_O_2_ works synergistically with other honey components, such as polyphenols, flavonoids, and methylglyoxal (MGO in Manuka honey), to enhance antimicrobial activity [53]. The combined action of these compounds results in the broad-spectrum antimicrobial effect of honey.

H_2_O_2_ in MNP honey ranged from 31.32 to 34.85 μg/100 g. Although difficult to compare due to the different analytical methodologies and units, Lehmann et al. [54] reported H_2_O_2_ in honey within the range of 0 to 283.2 μM (0 to 9633 μg/L). In another study, the levels of H_2_O_2_ were reported as 9.6 to 25.7 mM [55]. According to Lehmann et al. [54], glucose oxidase is inactive in fully ripened honey, but when honey is diluted, it converts β-d-glucose into H_2_O_2_ and d-gluconic acid. H_2_O_2_ production depends on the floral source, foraging time, honey age, storage conditions, dilution rate, and time since dilution. Optimal H_2_O_2_ accumulation occurs at 30–50% dilution, decreasing below 30% [54]. Variability may also be related to bee and hive health or environmental conditions. Other honey components, like catalase, can affect glucose oxidase activity [54].

Manuka honey is renowned for its effective antimicrobial properties despite having low H_2_O_2_ concentrations (not detected in this study’s sample), due to high levels of MGO [56]. MGO is a powerful antimicrobial agent derived from dihydroxyacetone in Manuka nectar [56]. Other compounds, such as leptosperin and flavonoids, further enhance its bioactivity [57]. The low H_2_O_2_ levels may also be due to higher catalase activity, which breaks H_2_O_2_ into water and oxygen. MGO’s stability ensures prolonged antimicrobial effects, making Manuka honey effective against various pathogens, even without high H_2_O_2_ levels [57].

### 3.5. In Vitro Antioxidant Activity

The antioxidant activity of honey samples has been determined using a diversity of well-established assays [29,31,32,36,38]. To correctly describe honey’s antioxidant activity, the present study employed three assays: the FRAP assay, based on the reduction of ferric TPTZ, and the DPPH^•^ and ABTS^•+^ assays, which measure the ability to scavenge radicals.

From the analysis in Table 4, it can be observed that all honey samples presented considerable antioxidant activity using all three assays. FRAP values for both campaigns ranged from 24.82 ± 0.51 to 84.73 ± 1.79 mg AAE/100 g honey. Except for the MNP2 sample from the 2nd campaign, all FRAP values from the 1st campaign were at least 1.5-fold higher than those from the 2nd sampling (FRAP mean value: 68.80 versus 33.10 mg AAE/100 g honey for the 1st and 2nd sampling). The mean values of the TPC and TFC assays were similar between the sampling campaigns. In the case of FRAP results, although the samples were collected from the same apiary, the year of sampling appears to influence the antioxidant activity of the honey samples. FRAP results suggest that, despite the similar amounts of phenolic compounds in both sampling campaigns, the type of bioactive compounds in each honey can vary greatly, consequently affecting the honey’s reducing activities.

Our results were consistent with those of Attanzio et al. [58], who analyzed thirty honeys from Western Sicily (Italy) and reported FRAP within 17.7 and 165 mg AAE/100 g. Some authors evaluated the reducing power of Portuguese honey samples [30,32,59]. However, most of them used the EC50 value to express the results, making it impossible to establish a comparison with our data. Furthermore, although Soares et al. [29] reported FRAP values in different units, a similar trend was observed in the present study with a higher correlation between TFC and FRAP results (R = 0.731) compared to TPC and FRAP results (R = 0.690) (Figure 1). These results suggest that TFC has a stronger influence on honey antioxidant activity than TPC.

The DPPH^•^ scavenging activity of honey samples ranged from 31.22 ± 0.50 mg TE/100 g honey (sample MNP6, 2nd campaign) to 84.65 ± 4.62 mg TE/100 g honey (sample MNP2, 2nd campaign). Regarding the ABTS assay, the minimum and maximum values were found for sample MNP8 in the 1st campaign (32.48 ± 0.39 mg TE/100 g honey) and sample MNP2 in the 2nd campaign (137.69 ± 0.98 mg TE/100 g honey), respectively. Despite all honey samples being collected in MNP, the apiaries were located in different areas, which can lead to significant differences in antioxidant activity [31]. In the study by Estevinho et al. [59], the antioxidant activity of 20 MNP honeys was determined by DPPH assay, and EC_50_ values were between 27.24 for dark honey and 68.17 mg/mL for clear honey. In the study by Ferreira et al. [32], the antioxidant activity of three uni-floral honey samples also from MNP was determined and ranged from 84.98 to 168.94 mg/mL. In both studies, dark honey exhibited higher antioxidant activity than clear honey. This difference was attributed to dark honey’s higher phenolic compound content, which is linked to their floral sources. Indeed, antioxidant production results from plants’ defence mechanism against environmental factors, such as ultraviolet radiation, temperature, and water stress. This explains why honey with the same botanical origin but collected from different apiary locations can show variations in antioxidant activity values [29]. Comparing these results with the literature, the DPPH^•^ values obtained for both campaigns were within the same range. Pedisić et al. [36] reported levels of 47.65 to 61.07 mg TE/100 g honey for mono-floral honeys collected in different geographical regions of Croatia. Zivkovic et al. [33] found that the DPPH capacity of Serbian honeys ranged from 11.26 to 68.83 mg TE/100 g honey. Attanzio et al. [58] reported DPPH values from 2.13 to 59.7 mg TE/100 g for Sicilian honeys. These findings highlight the huge diversity in antioxidant activity among honey samples.

For antioxidant activity measured by the ABTS assay, Pedisić et al. [36] reported higher values (147 to 167 mg TE/100 g honey) than the present study. In contrast, Attanzio et al. [58] reported a broader range (from 4.81 to 67.6 mg TE/100 g honey), closer to the present study’s values. This diversity of results can be attributed not only to the different botanical and geographical origins of the honey but also to variations in extraction conditions and techniques [13]. These factors can lead to differences in the phenolic compositions of honey extracts, as individual phenolic compounds have varying solubilities, affecting the extracts’ bioactivity [36]. Indeed, ABTS was strongly correlated with TPC (R = 0.959) and TFC (R = 0.969) values (Figure 2). Additionally, honey’s antioxidant capacity is also influenced by various constituents, such as peptides, organic acids, enzymes, Maillard reaction products, and other minor compounds, all of which contribute to its overall properties [36].

All samples from the 1st campaign could scavenge ^•^NO at a concentration of 2 mg/mL, although IC_50_ values could not be calculated at this concentration. In the 2nd campaign, only samples MNP1, MNP2, and MNP3 showed some degree of scavenging against this radical (Table 4). While the MNP samples did not show remarkable activity, they were more effective scavengers than Manuka honey, demonstrating only 7.72 ± 1.42% inhibition at the same concentration. Previous studies have shown that honey samples display some degree of O_2_^•−^ scavenging activity but are less active than their isolated phenolic extracts/fractions. For instance, IC_50_ values between 76.24 ± 0.55 and 116.48 ± 0.36 mg/mL for Moroccan *Euphorbia resinifera* and *Euphorbia officinarum* honey samples were reported, while IC_50_ values in the range of 3.64 ± 0.16 and 37.87 ± 0.24 mg/mL were obtained for their isolated phenolic fractions [60,61]. Similarly, Moroccan *Bupleurum spinosum* honey samples had high IC_50_ values between 75.75 ± 1.90 and 189.60 ± 0.43 mg/mL, demonstrating their weak ability to scavenge this radical [62]. On the other hand, a phenolic fraction obtained from a Portuguese *Calluna vulgaris* honey sample showed stronger activity (IC_50_= 0.181 ± 0.02 mg/mL) [63].

Honey samples were more active against O_2_^•−^, displaying IC_50_ values between 0.79 mg/mL (MNP2, 2nd campaign) and 1.76 mg/mL (MNP4, 1st campaign). Manuka honey was the least active sample. These results align with those obtained for Moroccan *Euphorbia resinifera* and *Euphorbia officinarum* honey samples (1.95 < IC_50_ < 4.26 mg/mL) [60]. In terms of ^•^OH scavenging, all samples from the 1st and 2nd campaigns were weak scavengers (≤20% at 2 mg/mL), a similar trend verified for Manuka honey. Nagai et al. [64] tested different honey samples (commercially available honey [Chinese milk vetch], pure honey [Chinese milk vetch], pure honey [acacia], mixed-breed honey, buckwheat honey, and honey [Japanese bee]) at a concentration of 50% (*v*/*v*) against ^•^OH and O_2_^•−^. They obtained percentages of inhibitions in the range of 70 and 90% against ^•^OH and from 20 to 100% against O_2_^•−^.

### 3.6. Enzyme Inhibition

Honey samples were tested against four enzymes, namely, acetylcholinesterase (AChE), butyrylcholinesterase (BuChE), tyrosinase, and α-glucosidase. At the highest concentration tested (2 mg/mL), none of the samples from the 1st and 2nd campaigns showed AChE inhibition. Concerning BuChE, only MNP4 (1st campaign) and MNP2, MNP3, and MNP4 honeys (2nd campaign) showed a weak degree of inhibition (≤20% at 2 mg/mL). Similar results were obtained for the Manuka sample, i.e., no inhibition against AChE and only 10% of BuChE inhibition at 2 mg/mL. These results are in accordance with those reported previously for 19 honey samples from varied botanical origin [5]. The highest AChE inhibition was observed in the case of thyme honey (21.17%) and the lowest AChE inhibition value was found for buckwheat honey (7.03%), while goldenrod and lavender honeys showed the highest (33.89%) and lowest (14.20%) BuChE inhibition, respectively.

Higher inhibitions were recorded for the other two enzymes (Table 4). α-Glucosidase was inhibited by all samples from the 1st and 2nd campaigns, with inhibitions ranging from 31.68 ± 4.90 to 49.30 ± 9.28% (1st campaign at 2 mg/mL) and from 38.05 ± 4.44 to 58.42 ± 7.79% (2nd campaign at 2 mg/mL). Tyrosinase was inhibited by all samples of the 1st campaign (11.72 ± 3.58–34.94 ± 4.91% at 2 mg/mL), while samples from the 2nd campaign were unable to inhibit this enzyme. While Manuka was not active against tyrosinase, at 2 mg/mL, it displayed comparable α-glucosidase inhibition to MNP samples (34.91 ± 8.47% at 2 mg/mL). Other authors have reported the weak activity of honeys against tyrosinase. *Arbutus unedo*, *Eucalyptus* spp., and *Cardus* f. honeys displayed IC_50_ values of 119.7 ± 5.2, 157.7 ± 2.5, and 64.3 mg/mL, respectively, which are concentrations much higher than the ones tested herein [4].

Concerning α-glucosidase inhibition, previous studies have described stronger activity for honeys and their phenolic fractions. For instance, Thummajitsakul et al. [65] reported IC_50_ values between 750 and 930 µg/mL for three honeys from stingless bees. Ali et al. [66] also evaluated the antidiabetic activity of stingless bee honey from different botanical origins. At the highest concentration tested (100 µg/mL), these honeys displayed the following α-glucosidase inhibitions: 38.75% (acacia), 68.32% (coconut), 67.51% (mangrove), 20.52% (startfruit), 45.52% (multifruit), 49.32% (multiflower), and 62.60% (tualang). Using a different approach, Zaidi et al. [67] evaluated the anti-AChE and anti-α-glucosidase potential of phenolic fractions isolated from 31 mono-floral and multifloral honeys from Algeria. AChE and α-glucosidase inhibition assays recorded IC_50_ values from 367 to 629 µg/mL and from 52.20 to 153.36 µg/mL, respectively.

### 3.7. Antimicrobial Activity

**Figure 4:**
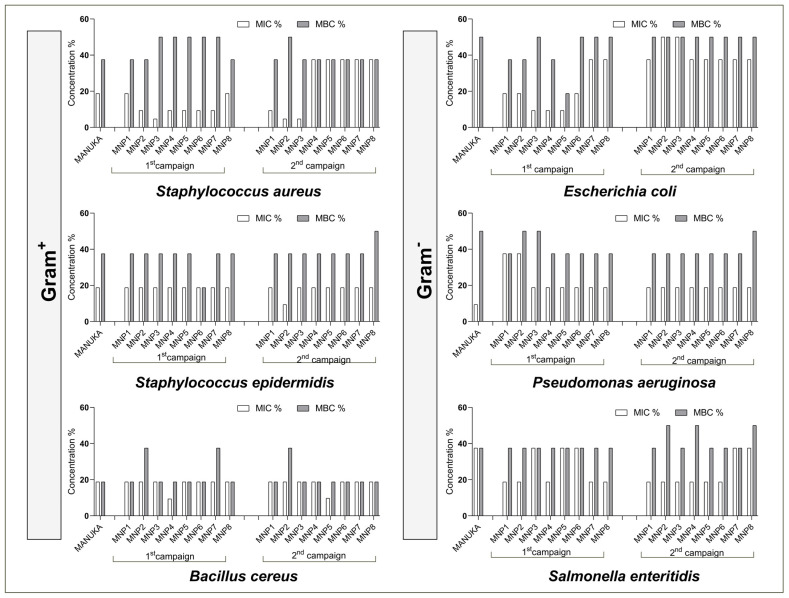
Antimicrobial activity of MNP and Manuka honeys.

MNP and Manuka honey samples were tested against three Gram+ (*Staphylococcus aureus*, *Staphylococcus epidermidis*, and *Bacillus cereus*) and three Gram− (*Escherichia coli*, *Pseudomonas aeruginosa*, and *Salmonella enteritidis*) bacteria. As can be seen in Figure 4, the most active samples against *S. aureus* were MNP3 (1st campaign) and MNP2 and MNP3 (2nd campaign) with MIC = 4.68%; MNP2 (2nd campaign) was the most effective against *S. epidermidis* (MIC = 9.38%); and MNP4 (1st campaign) and MNP5 (2nd campaign) presented an MIC of 9.37% for *B. cereus*.

For Gram− bacteria, MNP3, MNP4, and MNP5 (from the 1st campaign) were the most active against *E. coli* (MIC = 9.37%); all were moderately active against *P. aeruginosa* and *S. enteritidis* with MIC values between 18.75 and 37.50%. Manuka was only stronger than MNP samples when tested against *P. aeruginosa*, displaying an MIC value of 9.37%. According to Romário-Silva [68], the antimicrobial activity can be considered strong, moderate, and weak if MIC values are in the range of 1.0–12.5%, 12.5–50%, and >50%, respectively, demonstrating that MNP samples are strong antimicrobial agents.

The antimicrobial potential of honey is well described in the literature. Various factors contribute to its overall antimicrobial activity, including its low pH, high osmolarity, H_2_O_2_ content, presence of phenolic compounds and methylglyoxal, as well as bee-derived peptides like Defensin-1 [68,69]. Concerning Manuka honey, it is characterized by a high antibacterial activity even in the presence of catalase, which is an enzyme that breaks down H_2_O_2_. This non-peroxide activity is attributed to the high concentration of methylglyoxal (MGO), which is derived from dihydroxyacetone, present in large amounts in the nectar of *Leptospermum* species [70].

Matzen et al. [70] tested eleven Danish honey samples, plus one commercially processed culinary honey and two Manuka honeys against *Staphylococcus aureus* CCUG 1800, *Staphylococcus aureus* 1094-7, *Staphylococcus epidermidis* CCUG 39508, *Pseudomonas aeruginosa* SKN 1317, and *Escherichia coli* K 12. The authors verified great variation in different floral sources, with the Water Mint (*M. aquatica*), Linden (*T. cordata*), and Organic 2 (mixed organic flora) possessing the highest antibacterial activity on all the tested pathogens. Conversely, Manuka samples were not active against Gram− bacteria. Factors such as viscosity, osmolality, acidity, bioactive peptides, and H_2_O_2_ could influence the antibacterial activity of the Danish honeys.

## 4. Conclusions

The honey from MNP demonstrates high nutritional value and quality, with significant levels of essential amino acids and proline, as well as strong antioxidant and antimicrobial activities. These findings highlight the potential of this region to produce honey that meets or even exceeds the quality standards of renowned honey varieties like Manuka, reinforcing its status as a high-quality functional food product. The TPC in MNP honey ranged from 55.6 to 225 mg GAE/100 g, compared to 57.2 mg GAE/100 g for Manuka honey, with gallic acid, neochlorogenic acid, and catechin identified as key phenolic compounds. The obtained results suggest that future research could explore the prebiotic activity of MNP honey. Its phenolic compounds, which contribute to its antioxidant and antimicrobial properties, may also support gut health by selectively promoting beneficial bacteria while inhibiting harmful ones. Additionally, the observed variability across different collection spots shows the importance of environmental conditions and floral diversity in influencing honey composition, presenting opportunities for targeted honey production practices to maximize quality and nutritional benefits.

## Figures and Tables

**Table 1:** Total phenolic and flavonoid content, pH, and peroxide concentration of MNP honey samples and Manuka honey.

Parameter		MNP1	MNP2	MNP3	MNP4	MNP5	MNP6	MNP7	MNP8	Manuka
TPC (mg GAE/100 g)	1st campaign	68.72 ± 2.38 ***	72.77 ± 1.54 ****	71.01 ± 3.81 ****	71.66 ± 1.01 ****	64.17 ± 0.58 *	65.20 ± 1.09 *	70.21 ± 1.77 ****	55.58 ± 0.89 ns	57.16 ± 1.84
	2nd campaign	70.88 ± 0.64 ****	225.43 ± 1.89 ****	68.82 ± 4.23 ***	64.85 ± 0.52 *	62.16 ± 2.93 ns	67.23 ± 3.41 **	73.40 ± 2.14 ****	77.31 ± 2.80 ****	
TFC (mg EE/100 g)	1st campaign	7.24 ± 0.32 ****	6.86 ± 0.71 ****	7.74 ± 0.42 ****	4.33 ± 0.45 ns	4.71 ± 0.32 ns	4.25 ± 0.28 ns	4.47 ± 0.17 ns	4.37 ± 0.32 ns	3.52 ± 0.08
	2nd campaign	6.60 ± 0.27 ****	35.2 ± 0.6 ****	5.66 ± 0.27 ****	4.09 ± 0.08 ns	4.17 ± 0.58 ns	6.11 ± 0.17 ****	3.70 ± 0.18 ns	5.86 ± 0.52 ****	
pH	1st campaign	4.20	4.50	4.41	4.14	4.49	4.33	4.14	3.96	3.23
	2nd campaign	4.44	4.15	4.98	4.44	5.03	4.84	4.63	4.75	
H_2_O_2_ (μg/100 g)	1st campaign	32.23 ± 0.35 ****	34.62 ± 0.38 ****	34.49 ± 0.28 ****	32.04 ± 0.41 ****	33.37 ± 0.80 ****	33.81 ± 0.29 ****	33.56 ± 0.90 ****	31.32 ± 0.29 ****	0.00
	2nd campaign	33.03 ± 0.32 ****	33.18 ± 1.27 ****	34.85 ± 0.13 ****	31.32 ± 0.13 ****	34.02 ± 0.42 ****	33.56 ± 0.26 ****	34.59 ± 0.37 ****	33.29 ± 0.63 ****	

GAE—gallic acid equivalents; EE—epicatechin equivalents. For each parameter analyzed, samples from the 1st and 2nd campaigns were compared together with Manuka. ns—not significant differences (*p* > 0.05); statistically significant differences at *p* < 0.05 (*), *p* < 0.01 (**), *p* < 0.001 (***), and *p* < 0.0001 (****).

**Table 2:** Identification and quantification of phenolic compounds in different multifloral honey samples from MNP and Manuka honey analyzed by HPLC-DAD (results expressed in mg/100 g).

Compounds		MNP1	MNP2	MNP3	MNP4	MNP5	MNP6	MNP7	MNP8	Manuka
Naringin	1st campaign	0.56 ± 0.03 ****	0.51 ± 0.03 ****	0.45 ± 0.02 ****	0.15 ± 0.01 ****	0.44 ± 0.02 ****	0.31 ± 0.02 ****	0.25 ± 0.01 ****	0.08 ± 0.001 *	<LOD
	2nd campaign	1.67 ± 0.08 ****	<LOD ns	0.90 ± 0.05 ****	0.48 ± 0.02 ****	<LOD ns	0.32 ± 0.02 ****	<LOQ	0.70 ± 0.03 ****	
Kaempferol	1st campaign	0.17 ± 0.01 ****	0.20 ± 0.01 ****	<LOD ns	0.05 ± 0.00 ****	0.18 ± 0.01 ****	0.07 ± 0.00 ****	0.17 ± 0.01 ****	0.16 ± 0.01 ****	<LOD
	2nd campaign	0.19 ± 0.01 ****	<LOD ns	0.16 ± 0.01 ****	0.44 ± 0.02 ****	0.15 ± 0.01 ****	<LOD ns	0.19 ± 0.01 ****	<LOQ ns	
Naringenin	1st campaign	<LOQ ns	4.05 ± 0.20 ****	<LOD ns	2.46 ± 0.12 ****	<LOQ ns	<LOQ ns	2.72 ± 0.14 ****	0.11 ± 0.01 ns	<LOD
	2nd campaign	<LOD ns	5.60 ± 0.28 ****	0.05 ± 0.00 ns	0.10 ± 0.01 ns	<LOQ ns	0.13 ± 0.01 ns	0.02 ± 0.001 ns	0.16 ± 0.01 ****	
Quercetin-3-*O*-galactoside	1st campaign	3.21 ± 0.16 ****	1.20 ± 0.06 ns	6.53 ± 0.33 ****	1.66 ± 0.08 ns	<LOQ ns	2.62 ± 0.13 ****	1.62 ± 0.08 ns	5.84 ± 0.29 ****	1.29 ± 0.06
	2nd campaign	3.91 ± 0.20 ****	<LOD ns	1.39 ± 0.07 ns	2.57 ± 0.13 ****	4.02 ± 0.20 ****	<LOD ns	1.91 ± 0.10 ***	5.00 ± 0.25 ****	
Isorhamnetin-3-*O*-glucoside	1st campaign	ND ns	ND ns	ND ns	2.07 ± 0.10 ****	ND ns	ND ns	ND ns	ND ns	ND
	2nd campaign	ND ns	ND ns	ND ns	ND ns	ND ns	ND ns	ND ns	ND ns	
Isorhamnetin-3-*O*-rutinoside	1nd campaign	0.27 ± 0.01 ****	0.10 ± 0.00 ****	<LOD ns	0.13 ± 0.01 ****	0.15 ± 0.01 ****	0.21 ± 0.01 ****	<LOQ ns	0.18 ± 0.01 ****	<LOQ
	2nd campaign	0.20 ± 0.01 ****	<LOD ns	0.05 ± 0.00 ****	0.13 ± 0.01 ****	0.08 ± 0.00 ****	ND ns	<LOD ns	0.14 ± 0.01 ****	
Tiliroside	1st campaign	0.91 ± 0.05 ****	0.20 ± 0.01 ***	0.30 ± 0.01 ****	0.50 ± 0.02 ****	0.67 ± 0.03 ****	0.49 ± 0.02 ****	0.46 ± 0.02 ****	0.22 ± 0.01 ***	<LOQ
	2nd campaign	2.50 ± 0.12 ****	1.13 ± 0.06 ****	0.73 ± 0.04 ****	1.98 ± 0.10 ****	2.08 ± 0.10 ****	0.51 ± 0.03 ****	0.85 ± 0.04 ****	0.50 ± 0.03 ****	
Apigenin	1st campaign	0.49 ± 0.02 ****	0.28 ± 0.01 ****	<LOQ ns	0.06 ± 0.00 *	0.06 ± 0.00 *	0.48 ± 0.02 ****	0.26 ± 0.01 ****	0.22 ± 0.01 ****	<LOD
	2nd campaign	0.30 ± 0.01 ****	0.22 ± 0.01 ****	1.43 ± 0.07 ****	0.12 ± 0.01 ****	0.14 ± 0.01 ****	<LOD ns	0.93 ± 0.05 ****	<LOD	
Chrysin	1st campaign	0.82 ± 0.04 ****	0.56 ± 0.03 ****	0.41 ± 0.02 ****	0.05 ± 0.00 ns	0.29 ± 0.01 ****	0.60 ± 0.03 ****	1.31 ± 0.07 ****	0.22 ± 0.01 ****	<LOQ
	2nd campaign	0.74 ± 0.04 ****	<LOD ns	0.16 ± 0.01 ****	0.40 ± 0.02 ****	0.06 ± 0.00 ns	<LOQ ns	0.41 ± 0.02 ****	<LOQ ns	
Kaempferol-3-*O*-rutinoside	1st campaign	<LOQ ns	0.10 ± 0.00 ns	<LOQ ns	0.40 ± 0.02 ****	<LOQ ns	<LOD ns	<LOQ ns	<LOD ns	<LOD
	2nd campaign	1.04 ± 0.05 ****	<LOD ns	2.93 ± 0.15 ****	0.82 ± 0.04 ****	0.89 ± 0.04 ****	<LOD ns	<LOD ns	0.23 ± 0.01 ****	
Quercetin	1st campaign	0.50 ± 0.03 ****	1.12 ± 0.06 ****	<LOD ****	0.49 ± 0.02 ****	0.08 ± 0.001 ***	0.46 ± 0.02 ***	0.31 ± 0.02 ns	0.22 ± 0.01 ns	0.26 ± 0.01
	2nd campaign	2.79 ± 0.14 ****	0.97 ± 0.05 ****	0.64 ± 0.03 ****	0.25 ± 0.01 ns	<LOQ ****	0.60 ± 0.03 ****	1.67 ± 0.08 ****	0.95 ± 0.05 ****	
Catechin	1st campaign	6.99 ± 0.35 ****	4.29 ± 0.21 ****	5.37 ± 0.27 ****	6.30 ± 0.31 ****	6.48 ± 0.32 ****	5.35 ± 0.27 ****	4.81 ± 0.24 ****	7.41 ± 0.37 ****	1.21 ± 0.06
	2nd campaign	5.36 ± 0.27 ****	42.56 ± 2.13 ****	4.99 ± 0.25 ****	3.05 ± 0.15 ***	3.21 ± 0.16 ***	7.30 ± 0.36 ****	4.94 ± 0.25 ****	4.60 ± 0.23 ****	
Epicatechin	1st campaign	<LOD ****	0.06 ± 0.001 ****	<LOD ****	ND ****	1.22 ± 0.06 ns	0.18 ± 0.01 ****	4.15 ± 0.21 ****	0.33 ± 0.02 ****	1.33 ± 0.07
	2nd campaign	0.83 ± 0.04 ****	3.84 ± 0.19 ****	0.28 ± 0.01 ****	1.98 ± 0.10 ****	0.50 ± 0.03 ****	2.30 ± 0.12 ****	0.99 ± 0.05 ***	1.24 ± 0.06 ns	
Phloridzin	1st campaign	3.42 ± 0.17 ****	4.69 ± 0.23 ****	4.14 ± 0.21 ****	1.64 ± 0.08 ****	3.99 ± 0.20 ****	<LOD ****	2.81 ± 0.14 ****	1.34 ± 0.07 **	0.79 ± 0.04
	2nd campaign	3.10 ± 0.16 ****	4.82 ± 0.24 ****	5.25 ± 0.26 ****	3.77 ± 0.19 ****	3.89 ± 0.19 ****	4.13 ± 0.21 ****	2.64 ± 0.13 ****	3.37 ± 0.17 ****	
Phloretin	1st campaign	ND ns	ND ns	ND ns	ND ns	ND ns	ND ns	ND ns	ND ns	ND
	2nd campaign	<LOD ns	0.59 ± 0.03 ****	<LOD ns	<LOD ns	<LOD ns	0.12 ± 0.01 ***	<LOQ ns	<LOD ns	
Myricetin	1st campaign	0.86 ± 0.04 ns	0.73 ± 0.04 ns	0.52 ± 0.03 ****	0.80 ± 0.04 ns	0.67 ± 0.03 ns	0.48 ± 0.02 ****	0.34 ± 0.02 ****	0.31 ± 0.02 ****	0.77 ± 0.04
	2nd campaign	1.85 ± 0.09 ****	1.62 ± 0.08 ****	1.37 ± 0.07 ****	0.57 ± 0.03 ***	1.45 ± 0.07 ****	1.67 ± 0.08 ****	0.80 ± 0.04 ns	1.06 ± 0.05 ****	
Kaempferol-3-*O*-glucoside	1st campaign	ND ns	ND ns	ND ns	ND ns	ND ns	ND ns	ND ns	ND ns	ND
	2nd campaign	<LOD ns	ND ns	0.11 ± 0.01 ****	ND ns	0.07 ± 0.00 ***	0.36 ± 0.02 ****	0.51 ± 0.03 ****	0.05 ± 0.00 **	
Rutin	1st campaign	ND ns	ND ns	ND ns	ND ns	ND ns	ND ns	ND ns	ND ns	ND
	2nd campaign	0.88 ± 0.04 ****	2.72 ± 0.14 ****	1.87 ± 0.09 ****	2.51 ± 0.13 ****	1.49 ± 0.07 ****	0.95 ± 0.05 ****	1.23 ± 0.06 ****	0.99 ± 0.05 ****	
Gallic acid	1st campaign	5.49 ± 0.27 ****	5.69 ± 0.28 ****	5.89 ± 0.29 ****	9.68 ± 0.48 ****	5.32 ± 0.27 ****	5.25 ± 0.26 ****	7.28 ± 0.36 ****	8.61 ± 0.43 ****	37.14 ± 1.86
	2nd campaign	6.09 ± 0.30 ****	18.20 ± 0.91 ****	4.75 ± 0.24 ****	3.32 ± 0.17 ****	5.73 ± 0.29 ****	3.91 ± 0.20 ****	5.25 ± 0.26 ****	6.77 ± 0.34 ****	
Protocatechuic acid	1st campaign	1.97 ± 0.10 ****	1.69 ± 0.08 ****	1.82 ± 0.09 ****	3.40 ± 0.17 ****	1.76 ± 0.09 ****	1.58 ± 0.08 ****	2.89 ± 0.14 ****	1.43 ± 0.07 ****	0.14 ± 0.01
	2nd campaign	4.14 ± 0.21 ****	14.94 ± 0.75 ****	1.25 ± 0.06 ****	4.43 ± 0.22 ****	3.33 ± 0.17 ****	4.76 ± 0.24 ****	7.94 ± 0.40 ****	5.24 ± 0.26 ****	
Neochlorogenic acid	1st campaign	1.92 ± 0.10 ****	4.48 ± 0.22 ****	5.45 ± 0.27 ****	10.86 ± 0.54 ****	1.77 ± 0.09 ****	1.49 ± 0.07 ****	8.27 ± 0.41 ****	2.60 ± 0.13 ****	0.14 ± 0.01
	2nd campaign	2.07 ± 0.10 ****	14.77 ± 0.74 ****	2.06 ± 0.10 ****	5.04 ± 0.25 ****	2.85 ± 0.14 ****	5.52 ± 0.28 ****	4.16 ± 0.21 ****	4.70 ± 0.23 ****	
Caftaric acid	1st campaign	0.21 ± 0.01 ns	0.38 ± 0.02 ns	0.49 ± 0.02 ns	0.82 ± 0.04 ****	3.53 ± 0.18 ****	0.32 ± 0.02 ns	0.32 ± 0.02 ns	0.75 ± 0.04 ***	0.21 ± 0.01
	2nd campaign	0.46 ± 0.02 ns	9.49 ± 0.47 ****	0.31 ± 0.02 ns	0.20 ± 0.01 ns	0.39 ± 0.02 ns	0.18 ± 0.01 ns	0.20 ± 0.01 ns	0.78 ± 0.04 ****	
Chlorogenic acid	1st campaign	1.04 ± 0.05 ns	2.54 ± 0.13 ****	5.79 ± 0.29 ****	1.78 ± 0.09 **	1.03 ± 0.05 ns	0.98 ± 0.05 ns	5.71 ± 0.29 ****	1.07 ± 0.05 ns	0.90 ± 0.05
	2nd campaign	1.21 ± 0.06 ns	20.09 ± 1.00 ****	0.81 ± 0.04 ns	1.00 ± 0.05 ns	1.00 ± 0.05 ns	1.21 ± 0.06 ns	0.77 ± 0.04 ns	1.33 ± 0.07 ns	
4-*O*-caffeyolquinic acid	1st campaign	0.32 ± 0.02 ns	1.18 ± 0.06 ****	0.18 ± 0.01 ns	1.19 ± 0.06 ****	0.71 ± 0.04 ****	0.59 ± 0.03 **	4.71 ± 0.24 ****	1.11 ± 0.06 ****	0.33 ± 0.02
	2nd campaign	0.58 ± 0.03 **	2.39 ± 0.12 ****	0.91 ± 0.05 ****	0.47 ± 0.02 ns	0.58 ± 0.03 **	0.71 ± 0.04 ****	0.41 ± 0.02 ns	0.44 ± 0.02 ns	
Vanillic acid	1st campaign	4.10 ± 0.20 ****	7.51 ± 0.38 ****	2.03 ± 0.10 ****	3.30 ± 0.16 ****	2.83 ± 0.14 ****	2.84 ± 0.14 ****	1.26 ± 0.06 **	2.02 ± 0.10 ****	0.68 ± 0.03
	2nd campaign	8.20 ± 0.41 ****	7.54 ± 0.38 ****	0.95 ± 0.05 ns	1.49 ± 0.07 ***	0.48 ± 0.02 ns	0.83 ± 0.04 ns	0.52 ± 0.03 ns	0.56 ± 0.03 ns	
Caffeic acid	1st campaign	0.21 ± 0.01 ****	0.02 ± 0.001 ns	<LOD ns	0.09 ± 0.001 ***	0.19 ± 0.01 ****	0.40 ± 0.02 ****	0.11 ± 0.01 ****	0.10 ± 0.00 ****	<LOD
	2nd campaign	0.42 ± 0.02 ****	1.48 ± 0.07 ****	0.86 ± 0.04 ****	0.41 ± 0.02 ****	0.35 ± 0.02 ****	<LOD ns	<LOD ns	0.33 ± 0.02 ****	
Syringic acid	1st campaign	0.11 ± 0.01 ns	0.12 ± 0.01 ns	0.11 ± 0.01 ns	8.53 ± 0.43 ****	0.08 ± 0.001 ns	0.26 ± 0.01 ns	0.19 ± 0.01 ns	0.13 ± 0.01 ns	0.12 ± 0.01
	2nd campaign	0.20 ± 0.01 ns	3.17 ± 0.16 ****	0.50 ± 0.02 **	1.16 ± 0.06 ****	0.20 ± 0.01 ns	1.89 ± 0.09 ****	0.86 ± 0.04 ****	ND ns	
*p*-Coumaric acid	1st campaign	0.75 ± 0.04 ****	0.72 ± 0.04 ****	0.67 ± 0.03 ****	0.86 ± 0.04 ****	0.72 ± 0.04 ****	1.29 ± 0.06 ****	0.17 ± 0.01 **	0.08 ± 0.00 ns	<LOD
	2nd campaign	1.09 ± 0.05 ****	0.92 ± 0.05 ****	1.43 ± 0.07 ****	0.99 ± 0.05 ****	1.04 ± 0.05 ****	1.61 ± 0.08 ****	1.30 ± 0.07 ****	1.09 ± 0.05 ****	
*trans*-Ferulic acid	1st campaign	0.37 ± 0.02 ns	ND ****	ND ****	ND ****	ND ****	0.85 ± 0.04 ****	ND ****	0.18 ± 0.01 ****	0.39 ± 0.02
	2nd campaign	0.57 ± 0.03 ****	0.75 ± 0.04 ****	0.81 ± 0.04 ****	0.44 ± 0.02 ns	0.73 ± 0.04 ****	0.68 ± 0.03 ****	0.56 ± 0.03 ****	0.70 ± 0.04 ****	
Sinapic acid	1st campaign	2.32 ± 0.12 ****	1.72 ± 0.09 ****	1.27 ± 0.06 ****	ND ns	1.48 ± 0.07 ****	2.31 ± 0.12 ****	1.07 ± 0.05 ****	5.31 ± 0.27 ****	0.19 ± 0.01
	2nd campaign	ND ns	ND ns	ND ns	ND ns	ND ns	ND ns	ND ns	ND ns	
3,5-di-caffeoylquinic acid	1st campaign	0.16 ± 0.01 ****	0.08 ± 0.00 ***	<LOQ ns	0.42 ± 0.02 ****	<LOQ ns	<LOD ns	<LOQ ns	0.27 ± 0.01 ****	<LOQ
	2nd campaign	0.77 ± 0.04 ****	<LOD ns	0.48 ± 0.02 ****	0.28 ± 0.01 ****	0.90 ± 0.04 ****	0.31 ± 0.02 ****	0.53 ± 0.03 ****	0.30 ± 0.02 ****	
Ellagic acid	1st campaign	1.79 ± 0.09 ****	2.52 ± 0.13 ****	2.21 ± 0.11 ****	<LOD ns	1.99 ± 0.10 ****	<LOD ns	1.60 ± 0.08 ****	0.66 ± 0.03 ****	<LOQ
	2nd campaign	0.70 ± 0.03 ****	<LOD ns	0.61 ± 0.03 ****	0.23 ± 0.01 ***	<LOD ns	<LOD ns	ND ns	<LOD ns	
3,4-Di-caffeyolquinic acid	1st campaign	0.48 ± 0.02 ****	0.36 ± 0.02 ****	0.63 ± 0.03 ****	0.20 ± 0.01 ****	0.52 ± 0.03 ****	0.47 ± 0.02 ****	0.31 ± 0.02 ****	0.09 ± 0.00 *	<LOD
	2nd campaign	0.84 ± 0.04 ****	0.69 ± 0.03 ****	0.89 ± 0.04 ****	0.87 ± 0.04 ****	0.84 ± 0.04 ****	0.44 ± 0.02 ****	0.78 ± 0.04 ****	1.07 ± 0.05 ****	
Cinnamic acid	1st campaign	0.35 ± 0.02 ****	0.24 ± 0.01 ****	0.16 ± 0.01 ****	<LOD ns	0.20 ± 0.01 ****	0.15 ± 0.01 ****	0.06 ± 0.001 ****	0.13 ± 0.01 ****	ND
	2nd campaign	ND ns	ND ns	ND ns	ND ns	ND ns	ND ns	ND ns	ND ns	
*trans*-Epsilon viniferin	1st campaign	<LOD ns	0.05 ± 0.001 ****	<LOD ns	<LOD ns	<LOD ns	<LOD ns	<LOD ns	<LOD ns	<LOD
	2nd campaign	0.26 ± 0.01 ****	0.27 ± 0.01 ****	<LOD ns	0.26 ± 0.01 ****	<LOD ns	<LOD ns	<LOQ ns	<LOD ns	
Total	1st campaign	39.80	47.37	44.44	57.86	36.36	30.02	53.17	41.19	45.87
	2nd campaign	52.96	158.76	38.92	39.78	36.45	40.46	42.01	42.32	

LOD—limit of detection; LOQ—limit of quantification; ND—not detected; grey shadow—flavonoids; no shadow—phenolic acids and others. For each compound analyzed, samples from the 1st and 2nd campaigns were compared together with Manuka. ns—not significant differences (*p* > 0.05); statistical significant differences at *p* < 0.05 (*), *p* < 0.01 (**), *p* < 0.001 (***), and *p* < 0.0001 (****).

**Table 3:** Amino acids in different multifloral honey samples from MNP and Manuka honey analyzed by HPLC-FLD (results expressed in mg amino acid/100 g honey).

		MNP1	MNP2	MNP3	MNP4	MNP5	MNP6	MNP7	MNP8	Manuka
Asp	1st campaign	16.01 ± 0.05 ****	21.56 ± 0.44 ****	23.76 ± 0.23 ****	11.09 ± 0.29 ****	19.12 ± 0.43 ****	11.44 ± 0.72 ****	13.32 ± 0.18 ****	8.33 ± 0.01 ns	8.00 ± 0.14
	2nd campaign	13.49 ± 0.01 ****	6.42 ± 0.26 ****	13.60 ± 0.70 ****	5.64 ± 0.06 ****	9.95 ± 0.65 ****	7.03 ± 0.33 **	10.08 ± 0.62 ****	11.96 ± 0.48 ****	
Glu	1st campaign	14.12 ± 0.01 ****	20.46 ± 0.43 ****	22.71 ± 0.17 ****	12.54 ± 0.42 ****	19.79 ± 0.36 ****	10.76 ± 0.58 ns	12.72 ± 0.21 ****	6.78 ± 0.01 ****	9.73 ± 0.01
	2nd campaign	19.51 ± 0.17 ****	0.49 ± 0.01 ****	22.73 ± 1.30 ****	10.43 ± 0.04 ns	17.30 ± 0.89 ****	13.51 ± 0.74 ****	18.50 ± 1.25 ****	17.08 ± 0.97 ****	
Asn	1st campaign	7.04 ± 0.04 ****	6.57 ± 0.15 ****	7.35 ± 0.04 ****	7.11 ± 0.35 ****	5.29 ± 0.12 ****	2.28 ± 0.14 ****	4.08 ± 0.04 ****	3.84 ± 0.02 *	3.51 ± 0.02
	2nd campaign	8.05 ± 0.09 ****	1.32 ± 0.06 ****	6.70 ± 0.33 ****	2.22 ± 0.01 ****	5.54 ± 0.02 ****	3.31 ± 0.08 ns	4.98 ± 0.41 ****	8.14 ± 0.32 ****	
Ser	1st campaign	2.55 ± 0.03 ns	2.30 ± 0.13 ****	2.24 ± 0.01 ****	2.49 ± 0.03 ns	2.14 ± 0.01 ****	1.13 ± 0.09 ****	2.24 ± 0.03 ****	1.51 ± 0.06 ****	2.64 ± 0.06
	2nd campaign	2.41 ± 0.09 **	0.14 ± 0.01 ****	2.24 ± 0.21 ****	1.68 ± 0.12 ****	2.93 ± 0.16 ***	2.76 ± 0.13 ns	2.45 ± 0.17 *	1.67 ± 0.14 ****	
Gln	1st campaign	3.07 ± 0.02 ****	2.37 ± 0.01 ****	2.52 ± 0.17 ****	2.68 ± 0.01 ****	2.23 ± 0.09 ****	1.16 ± 0.10 ****	2.37 ± 0.01 ****	1.97 ± 0.06 ****	5.57 ± 0.01
	2nd campaign	9.20 ± 0.11 ****	0.06 ± 0.01 ****	6.47 ± 0.40 ****	4.09 ± 0.18 ****	6.01 ± 0.35 **	4.34 ± 0.26 ****	5.34 ± 0.40 ns	5.94 ± 0.26 *	
**His**	1st campaign	**1.30** ± 0.06 **	**1.24** ± 0.10 ****	**1.64** ± 0.14 **	**0.44** ± 0.04 ****	**1.48** ± **0.13** **ns**	**0.78** ± 0.04 ****	**0.35** ± 0.02 ****	**0.13** ± 0.01 ****	**1.47** ± **0.02**
	2nd campaign	**0.84** ± 0.06 ****	**0.32** ± 0.02 ****	**1.88** ± 0.15 ****	**0.28** ± 0.02 ****	**0.84** ± 0.04 ****	**0.38** ± 0.01 ****	**0.32** ± 0.02 ****	**0.44** ± 0.03 ****	
**Thr**	1st campaign	**0.31** ± 0.01 ****	**0.29** ± 0.01 ****	**0.29** ± 0.03 ****	**0.22** ± 0.02 ****	**0.09** ± 0.01 ****	**<LOD** ****	**0.06** ± 0.01 ****	**<LOD** ****	**1.04** ± **0.08**
	2nd campaign	**0.39** ± 0.01 ****	**<LOD** ****	**0.33** ± 0.01 ****	**0.04** ± 0.01 ****	**1.20** ± 0.10 ****	**0.99** ± **0.03** **ns**	**0.11** ± 0.01 ****	**0.06** ± 0.01 ****	
Tau	1st campaign	19.84 ± 0.01 ****	16.93 ± 0.20 ****	16.74 ± 0.69 ****	25.84 ± 1.36 ****	16.92 ± 0.38 ****	18.23 ± 1.08 ****	19.30 ± 0.16 ****	7.88 ± 0.28 ***	5.92 ± 0.38
	2nd campaign	15.56 ± 0.16 ****	2.48 ± 0.08 ****	10.21 ± 1.11 ****	6.34 ± 0.14 ns	15.76 ± 0.65 ****	7.71 ± 0.45 ***	10.08 ± 0.94 ****	7.54 ± 0.53 **	
Arg	1st campaign	6.13 ± 0.05 ****	6.11 ± 0.14 ****	6.19 ± 0.15 ****	5.71 ± 0.20 ****	6.03 ± 0.06 ****	4.78 ± 0.27 ****	5.58 ± 0.05 ****	4.01 ± 0.10 ****	2.70 ± 0.08
	2nd campaign	7.84 ± 0.007 ****	1.18 ± 0.03 ****	7.37 ± 0.48 ****	2.92 ± 0.08 ns	4.89 ± 0.12 ****	3.10 ± 0.14 *	4.93 ± 0.49 ****	4.91 ± 0.33 ****	
Ala	1st campaign	6.19 ± 0.09 ****	7.12 ± 0.10 ****	7.39 ± 0.14 ****	6.91 ± 0.21 ****	5.91 ± 0.01 ****	4.01 ± 0.20 ns	5.98 ± 0.17 ****	4.27 ± 0.07 **	3.83 ± 0.03
	2nd campaign	4.53 ± 0.01 ****	1.28 ± 0.03 ****	4.54 ± 0.20 ****	2.98 ± 0.02 ****	4.52 ± 0.34 ****	4.54 ± 0.34 ****	4.13 ± 0.36 ns	3.69 ± 0.21 ns	
Tyr	1st campaign	3.81 ± 0.02 ns	0.98 ± 0.02 ****	1.04 ± 0.04 ****	8.98 ± 0.33 ****	0.98 ± 0.02 ****	1.54 ± 0.06 ****	8.46 ± 0.12 ****	7.22 ± 0.02 ****	4.19 ± 0.18
	2nd campaign	9.28 ± 0.03 ****	1.18 ± 0.01 ****	2.23 ± 0.11 ****	4.17 ± 0.05 ns	1.99 ± 0.12 ****	2.85 ± 0.17 ****	20.91 ± 1.43 ****	3.00 ± 0.22 ****	
Cys	1st campaign	2.76 ± 0.01 ****	2.01 ± 0.08 ns	2.63 ± 0.25 ****	3.05 ± 0.16 ****	2.83 ± 0.03 ****	2.76 ± 0.11 ****	2.99 ± 0.21 ****	3.00 ± 0.34 ****	1.83 ± 0.02
	2nd campaign	3.73 ± 0.27 ****	2.30 ± 0.22 *	6.03 ± 0.30 ****	7.33 ± 0.31 ****	1.67 ± 0.04 ns	2.69 ± 0.19 ****	8.22 ± 0.52 ****	6.84 ± 0.26 ****	
Gly	1st campaign	<LOD ****	<LOD ****	<LOD ****	<LOD ****	<LOD ****	<LOD ****	<LOD ****	<LOD ****	0.70 ± 0.02
	2nd campaign	<LOD ****	<LOD ****	<LOD ****	<LOD ****	<LOD ****	<LOD ****	<LOD ****	<LOD ****	
**Val**	1st campaign	**1.96** ± **0.10** **ns**	**1.15** ± 0.07 ****	**1.70** ± 0.18 *	**2.17** ± 0.01 ****	**1.18** ± 0.10 ****	**0.83** ± 0.03 ****	**1.58** ± 0.01 ****	**1.39** ± 0.01 ****	**1.87** ± **0.01**
	2nd campaign	**2.42** ± 0.10 ****	**0.93** ± 0.02 ****	**1.96** ± **0.11** **ns**	**2.05** ± 0.06 *	**1.38** ± 0.08 ****	**1.51** ± 0.05 ****	**2.33** ± 0.11 ****	**2.13** ± 0.13 ***	
**Met**	1st campaign	<LOD ****	<LOD ****	<LOD ****	<LOD ****	<LOD ****	<LOD ****	<LOD ****	<LOD ****	**0.11** ± **0.01**
	2nd campaign	<LOD ****	<LOD ****	<LOD ****	<LOD ****	<LOD ****	<LOD ****	<LOD ****	<LOD ****	
B-Ala	1st campaign	<LOD ****	<LOD ****	<LOD ****	<LOD ****	<LOD ****	<LOD ****	<LOD ****	<LOD ****	10.76 ± 0.48
	2nd campaign	<LOD ****	<LOD ****	<LOD ****	<LOD ****	11.39 ± 0.72 ***	3.81 ± 0.13 ****	<LOD ****	<LOD ****	
**Lys**	1st campaign	**4.91** ± 0.18 ****	**12.89** ± 0.94 ****	**10.90** ± 1.17 ****	**2.59** ± 0.16 ****	**5.54** ± 0.35 ****	**2.08** ± 0.19 ****	**1.61** ± 0.04 ****	<LOD ****	**98.62** ± **4.95**
	2nd campaign	**0.98** ± 0.08 ****	**0.59** ± 0.06 ****	<LOD ****	<LOD ****	**23.98** ± 1.71 ****	**18.21** ± 1.01 ****	<LOD ****	<LOD ****	
**Trp**	1st campaign	**0.22** ± 0.01 ****	**0.32** ± 0.03 ****	**0.32** ± 0.01 ****	**0.11** ± 0.01 ****	**0.18** ± 0.01 ****	**0.10** ± 0.01 ****	**0.16** ± 0.01 ****	**0.17** ± 0.01 ****	**0.44** ± **0.01**
	2nd campaign	**1.13** ± 0.04 ****	**0.008** ± 0.0001 ****	**1.51** ± 0.10 ****	**0.23** ± 0.02 ****	**0.64** ± **0.02**	**0.21** ± 0.02 ****	**0.63** ± 0.01 ****	**0.68** ± 0.05 ****	
**Phe**	1st campaign	**8.84** ± 0.03 ****	**0.74** ± 0.04 ****	**0.94** ± 0.02 ****	**17.00** ± **0.58** **ns**	**1.45** ± 0.02 ****	**2.77** ± 0.19 ****	**14.73** ± 0.14 ****	**18.78** ± **0.10** **ns**	**17.83** ± **0.16**
	2nd campaign	**28.30** ± 0.38 ****	**2.93** ± 0.09 ****	**5.82** ± 0.28 ****	**21.28** ± 0.19 ****	**5.62** ± 0.30 ****	**11.64** ± 0.88 ****	**25.73** ± 1.83 ****	**11.06** ± 0.59 ****	
**Ile**	1st campaign	**0.87** ± 0.01 ****	**0.54** ± 0.01 ****	**0.58** ± 0.02 ****	**1.13** ± 0.06 *	**0.57** ± 0.02 ****	**0.43** ± 0.04 ****	**0.96** ± 0.03 ****	**0.78** ± 0.01 ****	**1.33** ± **0.08**
	2nd campaign	**1.67** ± 0.04 ****	**0.48** ± 0.02 ****	**1.10** ± 0.06 ****	**1.21** ± 0.02 ****	**0.84** ± 0.07 ****	**1.07** ± 0.09 ****	**1.32** ± **0.13** **ns**	**1.03** ± 0.08 ****	
**Leu**	1st campaign	**0.49** ± 0.02 ****	**0.37** ± 0.01 ****	**0.27** ± 0.01 ****	**0.75** ± 0.06 ****	**0.27** ± 0.02 ****	**0.26** ± 0.02 ****	**0.58** ± 0.01 ****	**1.02** ± 0.01 ****	**1.51** ± **0.09**
	2nd campaign	**1.25** ± 0.02 ****	**0.16** ± 0.01 ****	**0.65** ± 0.04 ****	**0.81** ± 0.07 ****	**0.53** ± 0.04 ****	**0.56** ± 0.05 ****	**0.80** ± 0.03 ****	**0.61** ± 0.04 ****	
Pro	1st campaign	102.20 ± 6.72 ****	72.01 ± 4.24 ****	85.12 ± 8.49 ****	155.10 ± 6.60 ****	98.92 ± 2.21 ****	97.30 ± 6.46 ****	115.17 ± 5.78 ****	74.79 ± 7.46 ****	43.42 ± 3.65
	2nd campaign	72.49 ± 2.28 ****	20.46 ± 1.42 ****	116.44 ± 3.82 ****	102.34 ± 1.99 ****	52.77 ± 3.56 *	47.66 ± 0.03 ns	135.36 ± 4.54 ****	94.31 ± 8.06 ****	
Total	1st campaign	202.63	175.95	194.36	265.93	190.93	162.66	212.26	145.87	227.04
	2nd campaign	203.08	42.73	211.81	176.04	169.72	137.89	256.25	181.12	
EEA	1st campaign	18.90	17.53	16.65	24.41	10.75	7.26	20.03	22.27	124.22
	2nd campaign	36.99	5.42	13.25	25.90	35.02	34.56	31.26	16.02	
NEEA	1st campaign	183.72	158.42	177.71	241.51	180.18	155.40	192.22	123.60	102.82
	2nd campaign	166.10	37.31	198.56	150.15	134.70	103.33	224.99	165.10	
EEA/Total%	1st campaign	9.33	9.96	8.57	9.18	5.63	4.46	9.44	15.26	54.71
	2nd campaign	18.21	12.68	6.26	14.71	20.63	25.06	12.20	8.85	
NEEA/Total %	1st campaign	90.67	90.04	91.43	90.82	94.37	95.54	90.56	84.74	45.29
	2nd campaign	81.79	87.32	93.74	85.29	79.37	74.94	87.80	91.15	
***Pro***/***Total*** %	1st campaign	**50.44**	**40.93**	**43.80**	**58.32**	**51.81**	**59.82**	**54.26**	**51.27**	**19.12**
	2nd campaign	**35.69**	**47.89**	**54.98**	**58.14**	**31.09**	**34.56**	**52.82**	**52.07**	

Bold: Essential amino acids. EAA—essential amino acids; NEAA—non-essential amino acids. For each aminoacid analyzed, samples from the 1st and 2nd campaigns were compared together with Manuka. ns—not significant differences (*p* > 0.05); statistically significant differences at *p* < 0.05 (*), *p* < 0.01 (**), *p* < 0.001 (***), and *p* < 0.0001 (****). Amino acids by elution order: Asp—aspartic acid; Glu—glutamic acid; Asn—asparagine; Ser—serine; Gln—glutamine; His—histidine; Thr—threonine; Arg—arginine; Ala—alanine; Tau—taurine; Tyr—tyrosine; Cys—cysteine; Gly—glycine; Val—valine; Met—methionine; Lys—lysine; Trp—tryptophan; Phe—phenylalanine; Ile—isoleucine; Leu—leucine; Hyp—hydroxyproline; Pro—proline.

**Table 4:** In vitro antioxidant activity and enzyme inhibition activity of MNP honey samples and Manuka honey.

Parameter		MNP1	MNP2	MNP3	MNP4	MNP5	MNP6	MNP7	MNP8	Manuka
		Antioxidant activity								
FRAP (mg AAE/100 g honey)	1st campaign	73.24 ± 3.85 ****	81.73 ± 2.36 ****	84.73 ± 1.79 ****	53.13 ± 1.66 ****	70.95 ± 2.69 ****	64.28 ± 1.10 ****	68.47 ± 2.36 ****	53.96 ± 2.34 ****	24.59 ± 1.27
	2nd campaign	38.49 ± 0.30 ****	128.11 ± 2.85 ****	38.56 ± 0.48 ****	33.72 ± 0.33 ***	34.13 ± 1.06 ***	24.82 ± 0.51 ns	26.46 ± 0.55 ns	35.49 ± 0.21 ****	
DPPH (mg TE/100 g honey)	1st campaign	51.78 ± 2.78 ****	60.35 ± 2.15 ****	57.89 ± 2.36 ****	36.02 ± 0.11 ****	49.53 ± 3.77 ****	44.29 ± 0.72 ****	42.01 ± 0.67 ****	37.46 ± 0.55 ****	98.65 ± 2.23
	2nd campaign	56.81 ± 2.93 ****	84.65 ± 4.62 ****	47.25 ± 1.37 ****	32.54 ± 1.26 ****	33.75 ± 2.17 ****	31.22 ± 0.50 ****	40.85 ± 0.54 ****	39.01 ± 1.96 ****	
ABTS (mg TE/100 g honey)	1st campaign	48.03 ± 1.21 ****	53.57 ± 0.63 ****	51.08 ± 1.06 ****	36.57 ± 1.21 ***	48.42 ± 1.80 ****	42.90 ± 1.73 ****	41.55 ± 1.17 ****	32.48 ± 0.39 ns	29.99 ± 0.62
	2nd campaign	35.40 ± 1.89 **	137.69 ± 0.98 ****	52.98 ± 0.81 ****	43.38 ± 0.66 ****	44.11 ± 1.21 ****	48.61 ± 1.34 ****	32.63 ± 0.57 ns	52.32 ± 2.18 ****	
		Antiradical activity								
•NO scavenging activity (% at 2 mg/mL)	1st campaign	32.38 ± 5.02 ****	33.54 ± 0.64 ****	33.62 ± 6.12 ****	24.60 ± 3.39 ****	31.58 ± 0.56 ****	30.85 ± 2.58 ****	26.42 ± 2.05 ****	15.93 ± 0.69 **	7.72 ± 1.42
	2nd campaign	15.68 ± 0.69 **	18.56 ± 3.13 ***	30.13 ± 0.87 ****	7.33 ± 1.41 ns	n.a.	n.a.	n.a.	4.31 ± 0.90 ns	
•OH scavenging activity (% at 2 mg/mL)	1st campaign	8.06 ± 0.95 ns	15.02 ± 1.16 ****	12.35 ± 1.20 ****	9.91 ± 0.37 **	8.74 ± 0.82 ns	8.58 ± 0.84 ns	13.96 ± 0.66 ****	9.37 ± 0.41 *	6.90 ± 0.88
	2nd campaign	9.37 ± 0.41 *	13.87 ± 0.28 ****	9.96 ± 0.72 **	6.67 ± 0.73 ns	9.13 ± 0.65 *	6.84 ± 0.92 ns	8.56 ± 1.56 ns	9.37 ± 0.41 ns	
O2^•−^ scavenging activity (% at 2 mg/mL)/ [IC_50_, mg/mL]	1st campaign	64.98 ± 0.68 ****/[1.74]	82.61 ± 0.38 ****/[1.33]	79.55 ± 1.51 ****/[1.36]	66.93 ± 1.06 ****/[1.76]	77.32 ± 0.81 ****/[1.58]	66.09 ± 1.73 ****/[1.74]	70.63 ± 3.33 ****/[1.56]	69.38 ± 2.28 ****/[1.51]	17.30 ± 7.20/[>2.00]
	2nd campaign	62.80 ± 4.69 ****/[1.69]	84.59 ± 1.35 ****/[0.79]	65.48 ± 4.56 ****/[1.18]	64.64 ± 2.77 ****/[1.61]	66.39 ± 1.24 ****/[1.46]	60.27 ± 3.40 ****/[1.67]	66.32 ± 6.11 ****/[1.61]	68.48 ± 0.33 ****/[1.56]	
		Enzymatic inhibitory activity								
Tyrosinase inhibition (% at 2 mg/mL)	1st campaign	34.94 ± 4.91 ****	19.05 ± 3.04 ****	19.80 ± 1.89 ****	17.53 ± 1.18 ****	16.19 ± 1.57 ****	21.82 ± 2.25 ****	11.72 ± 3.58 ***	17.38 ± 2.89 ****	n.a.
	2nd campaign	n.a.	n.a.	n.a.	n.a.	n.a.	n.a.	n.a.	n.a.	
α-Glucosidase inhibition (% at 2mg/mL)	1st campaign	39.38 ± 10.10 ns	43.18 ± 9.14 ns	38.18 ± 3.55 ns	48.28 ± 10.32 ns	40.67 ± 8.08 ns	31.68 ± 4.90 ns	49.30 ± 9.28 ns	46.24 ± 1.00 ns	34.91 ± 8.47
	2nd campaign	58.42 ± 7.79 **	38.05 ± 4.44 ns	41.47 ± 6.93 ns	39.48 ± 3.40 ns	43.91 ± 5.02 ns	55.51 ± 6.25 **	53.16 ± 4.82 *	40.48 ± 2.81 ns	

AAE—ascorbic acid equivalents; ABTS—2,2′-azinobis [3-ethyl-2,3-dihydrobenzothiazole-6-sulphonate; DPPH—2,2-Diphenyl-1-picrylhydrazyl; FRAP—ferric-reducing activity of plasma; ^•^NO—nitric oxide radical; ^•^OH—hydroxyl radical; O_2_^•−^—super oxide radical; TE—trolox equivalents; n.a.—not active. For each parameter analyzed, samples from the 1st and 2nd campaigns were compared together with Manuka. ns—not significant differences (*p* > 0.05); statistically significant differences at *p* < 0.05 (*), *p* < 0.01 (**), *p* < 0.001 (***), and *p* < 0.0001 (****).

## Data Availability

The data presented in this study are available on request from the corresponding author. The data are not publicly available due to privacy restrictions.

## Supplementary Materials

The following supporting information can be downloaded at: https://www.mdpi.com/article/10.3390/foods14060963/s1, standards used for phenolic compounds analysis; standards used for amino acid analysis.; Table S1: Honey samples from MNP analyzed in the current study; Table S2: Calibration data used for quantifying individual phenolic compounds in honey samples from Montesinho Natural Park; Table S3: Calibration data used for quantifying individual amino acids in honey samples from Montesinho Natural Park.
