# Supplementary material for: Exploring the Nutritional Value and Health Benefits of Honey from the Portuguese Protected Area of Montesinho Natural Park
Source: Foods. 2025 Mar 12;14(6):963. doi: 10.3390/foods14060963 (PMC11941713; doi:10.3390/foods14060963)
Supplement: Supplementary file 1 [file foods-14-00963-s001.zip › foods-3485328-supplementary.pdf]

Supplementary material

Exploring the Nutritional Value and Health Benefits of Honey from the Portuguese Protected Area of Montesinho Natural Park

Clara Grosso[1,*], Sónia Soares[1], Aurora Silva[1,2], Cristina Soares[1], Manuela M. Moreira[1], Olena Dorosh[1], Fátima Barroso[1], Miguel A. Prieto[2], Cristina Delerue-Matos[1]

[a] *REQUIMTE/LAQV, Instituto Superior de Engenharia do Porto, Instituto Politécnico do Porto, Rua Dr. António Bernardino de Almeida 431, 4249-015 Porto, Portugal*

[b] *Universidade de Vigo, Nutrition and Food Group (NuFoG), Department of Analytical Chemistry and Food Science, Instituto de Agroecoloxía e Alimentación (IAA) – CITEXVI, 36310 Vigo, Spain.*

* Corresponding author

Email address: claragrosso@graq.isep.ipp.pt

Standards used for phenolic compounds analysis.

GA (≥99%), protocatechuic acid (99.63%), neochlorogenic acid (≥98%), caftaric acid (≥97%), chlorogenic acid (> 95%), 4-caffeyolquinic acid (≥98%), vanillic acid (≥97%), caffeic acid (≥98%), syringic acid (≥98%), *p*-coumaric acid (≥98%), *trans*-ferulic acid (≥99%), sinapic acid (≥99%), 3,5-di-*O*-caffeyolquinic acid (≥95%), ellagic acid (≥95%), 4,5-di-*O*-caffeyolquinic acid (≥90%), cinnamic acid (≥99%); flavonoids: (+)-catechin (≥98%), (-)-epicatechin (≥90%), naringin (≥95%), quecetin-3-*O*-galactoside (≥97%), rutin hydrate (≥94%), myricetin (≥96%), kaempferol-3-*O*-glucoside (≥95%), kaempferol-3-*O*-rutinoside (≥98%), isorhamnetin-3-*O*-glucoside (≥98%), isorhamnetin-3-*O*-rutinoside (≥99%), naringenin (98%), quercetin (95%), kaempferol (≥98%), apigenin (≥99%), chrysin (≥99%), tiliroside (≥98%); chalcones: phloridzin dehydrate (99%) and phloretin (≥98.5%); stilbenoids: *trans*-epsilon viniferin (≥95%).

Standards used for amino acid analysis.

Aspartic acid (Asp), arginine (Arg), asparagine (Asn), β-alanine (β-ala), cysteine (Cys), histidine (His), isoleucine (Ile), leucine (Leu), lysine (Lys), methionine (Met), norvaline (Nor), proline (Pro), serine (Ser), taurine (Tau), threonine (Thr), tyrosine (Tyr) and valine (Val) are all from Sigma-Aldrich. Alanine (Ala), glutamic acid (Glu), glycine (Gly) and phenylalanine (Phe) were from Merck, while glutamine (Gln) and tryptophan (Trp).

Table S1. Honey samples from MNP analyzed in the current study.

Apiary		Honey samples	
Name	Coordinates	2021(1st campaign)	2023(2nd campaign)
Lindolfo	41°50' 37.9"N 6°55' 44.9"W	MNP1	MNP1
Pinela	41°53' 21.1"N 6°49' 43.1"W	MNP2	MNP2
Merize	41°53'50"N 6°54'19"W	MNP3	MNP3
Cabanelas	41°56'22"N 7°03'57"W	MNP4	MNP4
Vale de Cavagem	41°50"34.9'N; 6°57"18.6'W	MNP5	MNP5
Nuzedo de Cima	41°53'30"N 7°04'30"W	MNP6	MNP6
Quiraz	41°55'45"N 7°09'53"W	MNP7	MNP7
Guadramil	41°54'53"N 6°34'01"W	MNP8	MNP8

Table S2. Calibration data used for quantifying individual phenolic compounds in honey samples from Montesinho Natural Park

Compounds	(m ± Δm)[a]	(b ± Δb)[b]	r^2	LOD[c] (mg/L)	LOQ[d] (mg/L)
**Phenolic acids**					
Gallic acid	54220 ± 97	57.7 ± 6	0.9999	0.120	0.400
Protocatechuic acid	32064 ± 24	1024 ± 477	0.9999	0.104	0.346
Neochlorogenic acid	50502 ± 344	5751 ± 288	0.9998	0.449	1.495
Caftaric acid	18700 ± 50	966 ± 48.3	0.9999	0.177	0.591
Chlorogenic acid	27244 ± 57	-1445 ± 144	0.9999	0.292	0.972
4-*O*-caffeyolquinic acid	12334 ± 202	-1032 ± 52	0.9995	0.190	0.632
Vanillic acid	36254 ± 55	1215 ± 121	0.9999	0.215	0.715
Caffeic acid	69190 ± 45	124 ± 12	0.9999	0.090	0.302
Syringic acid	61262 ± 72	2088 ± 209	0.9999	0.165	0.551
p-coumaric acid	146520 ± 1176	-33763 ± 3376	0.9991	1.700	5.680
Ferulic acid	61448 ± 39	1501.6 ± 789	0.9999	0.089	0.298
Sinapic acid	29742 ± 16	124 ± 12	0.9999	0.075	0.250
3,5-di-caffeoylquinic acid	62289 ± 829	-11003 ± 3153	0.9991	0.341	1.130
Ellagic acid	44947 ± 1040	-30767 ± 20152	0.9973	3.090	10.30
3,4-di-*O*-caffeoylquinic acid	28503 ± 410	32 ± 3	0.9991	0.383	1.270
Cinnamic acid	183447 ± 103	2472 ± 247	0.9999	0.079	0.263
**Flavonoids**					
Quercetin-3-*O*-galactoside	23597 ± 27	466 ± 47	0.9999	0.159	0.531
Myricetin	27506 ± 260	-12304 ± 5242	0.9950	1.320	4.430
Catechin	13826 ± 56	3021 ± 1127	0.9999	0.568	1.890
Epicatechin	15117 ± 60	1178 ± 118	0.9999	0.558	1.860
Quercetin-3-*O*-glucopyranoside	48964 ± 119	17.0 ± 0.9	0.9999	0.161	0.535
Quercitrin	32796 ± 199	2974 ± 149	0.9999	0.399	1.331
Tiliroside	67386 ± 95	-1771 ± 177	0.9999	0.198	0.658
Kaempferol	33473 ± 102	-3301 ± 330	0.9999	0.430	1.43
Kaempferol-3-*O*-glucoside	22171 ± 45	792 ± 79	0.9999	0.286	0.955
Isorhamnetin-3-O-glucoside	31445 ± 101	2000 ± 100	0.9999	0.211	0.702
Kaempferol-3-*O*-rutinoside	24228 ± 55	-1094 ± 109	0.9999	0.319	1.06
Isorhamnetin-3-O-rutinoside	23574 ± 115	2374 ± 119	0.9999	0.323	1.075
Quercetin	30017 ± 212	-7386 ± 739	0.9997	0.992	3.30
Apigenin	100189 ± 353	-7277 ± 727	0.9999	0.495	1.65

**Compounds**	**(m ± Δm)[a]**	**(b ± Δb)[b]**	**r^2**	**LOD[c] (mg/L)**	**LOQ[d] (mg/L)**
Chrysin	88535 ± 143	-3131 ± 313	0.9999	0.226	0.755
trans-e-viniferin	61705 ± 529	-1021 ± 51	0.9997	0.564	1.88
Naringin	36536 ± 25	1384 ± 499	0.9999	0.095	0.317
Naringenin	68101 ± 90	-2516 ± 1813	0.9999	0.186	0.619
trans-polydatin	64539 ± 221	4280 ± 214	0.9999	0.226	0.753
Rutin	83207 ± 51	1937 ± 1034	0.9999	0.087	0.289
Resveratrol	181186 ± 1303	-16059 ± 803	0.9998	0.474	1.570
**Others**					
Phloridzin	47726 ± 42	78 ± 8	0.9999	0.125	0.416
Phloretin	71734 ± 154	84 ± 8	0.9999	0.302	1.01

Table S3. Calibration data used for quantifying individual amino acids in honey samples from Montesinho Natural Park.

AA	m ± Δm	b ±Δb	R^2	LOD (µg/L)	LOQ (µg/L)
Asp	1339825 ± 9216	46843 ± 922	0.9998	0.32	1.07
Glu	1268261 ± 7190	41527 ± 719	0.9998	0.34	1.13
Asn	1555625 ± 2743	89268 ± 724	0.9999	0.28	0.92
Ser	1658915 ± 7137	327468 ± 714	0.9999	0.26	0.86
Gln	1605555 ± 2580	40347 ± 260	0.9999	0.27	0.89
His	466680 ± 9239	195189 ± 1012	0.9995	0.92	3.06
Thr	1134823 ± 7924	759758 ± 8276	0.9979	0.38	1.26
Arg	1083638 ± 4453	38851 ± 856	0.9999	0.40	1.32
Ala	2053198 ± 9540	282953 ± 954	0.9999	0.21	0.70
Tau	400122 ± 6184	128635 ± 618	0.9988	1.07	3.57
Tyr	1094272 ± 8106	-11978 ± 811	0.9997	0.39	1.31
Cys	215032 ± 25550	124607 ± 687	0.9862	1.99	6.65
Gly	698516 ± 7416	348497 ± 740	0.9994	0.61	2.05
Val	2396286 ± 2560	370049 ± 260	0.9999	0.18	0.60
Met	1399713 ± 5022	20822 ± 500	0.9999	0.31	1.02
Lys	18751 ± 1198	18702 ± 186	0.9939	22.90	76.20
Trp	970928 ± 2810	47095 ± 281	0.9999	0.44	1.47
Phe	1285043 ± 4143	336 ± 410	0.9999	0.33	1.11
Ile	1912716 ± 5865	126941 ± 587	0.9999	0.22	0.75
Leu	1895411 ± 9276	24107 ± 899	0.9999	0.23	0.75
Hyp	32831 ± 1948	-16405 ± 255	0.9979	13.10	43.50
Pro	27651 ± 1566	10526 ± 1917	0.9905	15.50	51.70

LOD=(3×SDblank)/m and LOQ=(10×SDblank)/m, where SDblank represents the stand-ard deviation of the blank, and m is the slope of the straight-line equation. Aminoacids by elution order: Asp – Aspartic acid; Glu – Glutamic acid; Asn – Asparagine; Ser – Serine; Gln – Glutamine; His – Histidine; Thr – Threonine; Arg – Arginine; Ala – Alanine; Tau – Taurine; Tyr – Tyrosine; Cys – Cysteine; Gly – Glycine; Val – Valine; Met – Methionine; Lys – Lysine; Trp – Tryptophan; Phe – Phenylalanine; Ile – Isoleucine; Leu – Leucine; Hyp – Hydroxyproline; Pro – Proline.
